# Motif-VI loop acts as a nucleotide valve in the West Nile Virus NS3 Helicase

**DOI:** 10.1093/nar/gkae500

**Published:** 2024-06-17

**Authors:** Priti Roy, Zachary Walter, Lauren Berish, Holly Ramage, Martin McCullagh

**Affiliations:** Department of Chemistry, Oklahoma State University, Stillwater, OK 74078, USA; Department of Microbiology and Immunology, Thomas Jefferson University, Philadelphia, PA 19107, USA; Department of Microbiology and Immunology, Thomas Jefferson University, Philadelphia, PA 19107, USA; Department of Microbiology and Immunology, Thomas Jefferson University, Philadelphia, PA 19107, USA; Department of Chemistry, Oklahoma State University, Stillwater, OK 74078, USA

## Abstract

The *Orthoflavivirus* NS3 helicase (NS3h) is crucial in virus replication, representing a potential drug target for pathogenesis. NS3h utilizes nucleotide triphosphate (ATP) for hydrolysis energy to translocate on single-stranded nucleic acids, which is an important step in the unwinding of double-stranded nucleic acids. Intermediate states along the ATP hydrolysis cycle and conformational changes between these states, represent important yet difficult-to-identify targets for potential inhibitors. Extensive molecular dynamics simulations of West Nile virus NS3h+ssRNA in the apo, ATP, ADP+P_i_ and ADP bound states were used to model the conformational ensembles along this cycle. Energetic and structural clustering analyses depict a clear trend of differential enthalpic affinity of NS3h with ADP, demonstrating a probable mechanism of hydrolysis turnover regulated by the motif-VI loop (MVIL). Based on these results, MVIL mutants (D471L, D471N and D471E) were found to have a substantial reduction in ATPase activity and RNA replication compared to the wild-type. Simulations of the mutants in the apo state indicate a shift in MVIL populations favoring either a closed or open ‘valve’ conformation, affecting ATP entry or stabilization, respectively. Combining our molecular modeling with experimental evidence highlights a conformation-dependent role for MVIL as a ‘valve’ for the ATP-pocket, presenting a promising target for antiviral development.

## Graphical Abstract

**Graphical Abstract:**
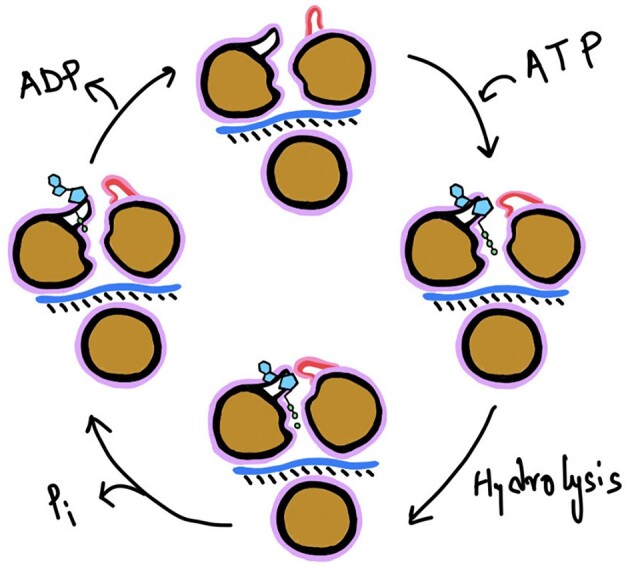

## Introduction

West Nile virus (WNV), a member of the *Orthoflavivirus* genus, is a global threat to human health and has become endemic in many areas of the world (1,2). Within the USA, WNV is designated as one of the most important zoonotic diseases (1,2). This virus is referred to as a re-emerging pathogenic virus, usually amplified in a mosquito-bird-mosquito enzootic transmission cycle (3). However, WNV can infect dead-end hosts, such as humans and horses, causing disease ranging from mild febrile illness to severe neurological disease. Human-human transmission is also reported, for example, through blood transfusion (4–6). Currently, there are no FDA-approved vaccines for humans to prevent WNV infection. While a handful of promising human vaccines have been tested, significant barriers exist in advancing these candidates to phase 3 clinical trials (7–9). Thus, the continued development of specific antiviral therapeutics to treat WNV disease is critical. These efforts necessitate a detailed molecular understanding of essential viral protein functions to identify antiviral targets.

The C-terminal end of the non-structural protein 3 (NS3) marks a promising antiviral target due to its pivotal role in viral replication. This region of NS3 functions as a DEAH-box helicase (NS3h) belonging to superfamily-2 (SF2) and is a structurally conserved monomeric protein in the *Orthoflavivirus* genus. This protein is involved in a critical step of replication: unwinding the double-stranded RNA (ds-RNA) replication intermediate by hydrolyzing the nucleotide triphosphate (NTP) in its active site (10). Several studies have reported that increased virulence of WNV strains is due to mutations in the NS3 helicase (NS3h) (11–14). WNV NS3h also performs nucleoside 5′-triphosphatase (NTPase) and 5′-terminal RNA triphosphatase (RTPase) activities but displays a strong preference for ATP as an NTPase substrate (15). As the binding site of RNA (RNA-cleft) and ATP (ATP-pocket) are within a single monomeric form, it is important to elucidate the structural details of NS3h to understand its function for structure-specific inhibitor design.

**Figure 1.**
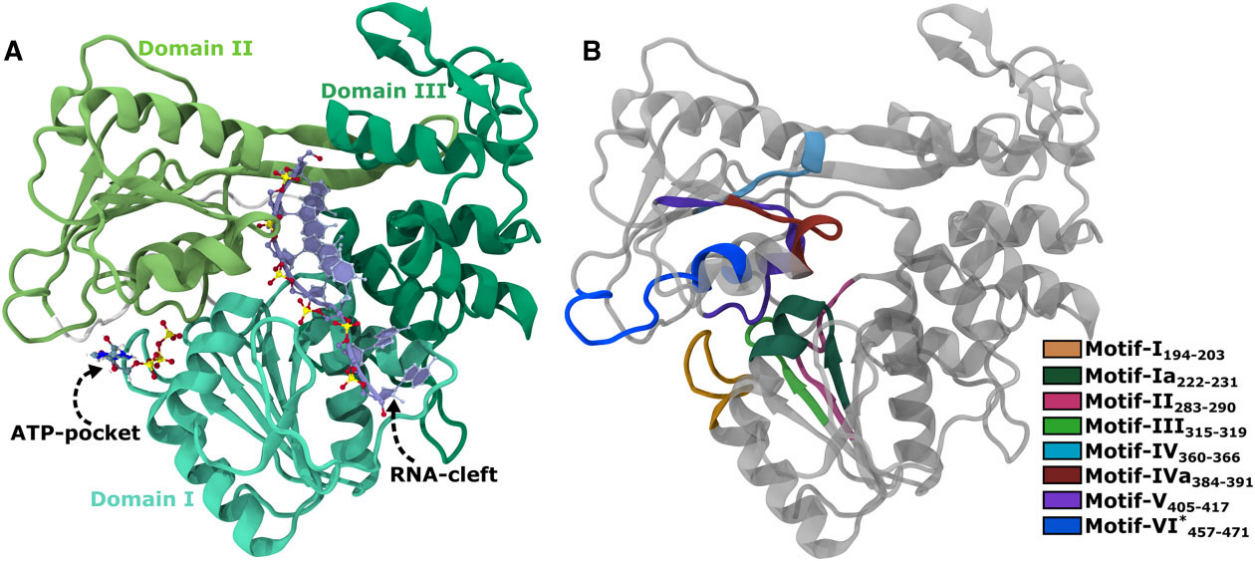
Structural representation of WNV NS3 helicase (NS3h). (**A**) The NS3h structure is displayed in cartoon mode marking three domains with different shades of green color. An ATP-pocket filled with ATP is located at the interface of Domain I and Domain II; the RNA-cleft filled with ssRNA is located at the interface of Domain III with Domain I and Domain II. (**B**) Conserved structural motifs are shown here with distinct color code. For clarity, the remaining regions of protein are colored in grey along with removal of ATP and ssRNA. This is an initial modelled structure using DENV4 (PDB ID: 2JLV (20)) and the entire Figure prepared by VMD (21).

The RNA-cleft and ATP-pocket in NS3h are distal and yet functionally correlated. NS3h spans amino acid residues 180 to 619 and adopts a tertiary structure comprised of three domains. Figure 1 provides a graphical illustration elucidating the NS3h structure and its binding sites. Domains I and II are primarily characterized by β-strands, sharing the Rossman’s fold, and together they constitute the ATP-pocket at the interface. In contrast, domain III is predominantly composed of α-helices and forms the RNA-cleft along the edges of the other two domains. As in other SF2 helicases, WNV NS3h consists of eight sequence-conserved motifs: motifs **I**, **II**, **III** and **VI** are involved in ATPase activity, while motifs **Ia**, **IV** and **IVa** interact with RNA, and motif **V** (16) is related to both functions. WNV NS3h can perform ATP hydrolysis and RNA binding independently (15,17), but the presence of RNA stimulates ATP hydrolysis, and the unwinding of double-stranded RNA (dsRNA) is an ATP hydrolysis-dependent process (18,19). These results indicate that the unwinding process is fueled by the free energy generated through the ATP hydrolysis cycle.

Translocation is an elementary step in NS3h-mediated unwinding, and its progression is marked by changing affinity for hydrolysis substrate and product. However, the dsRNA unwinding mechanism of the *Orthoflavivirus* NS3h remains elusive. A compilation of studies suggest that a small-step, ATP-dependent, recurring translocation cycle leads the protein to move unidirectionally from the 3′ to 5′ end by alternating strong and weak binding to ssRNA (22–25). The accumulation of these small steps results in the protein traversing a long stretch of ssRNA, leading to dsRNA unwinding. Experimental studies have observed that ATP binding induces a conformational change in the protein by closing Domain I and Domain II, causing the forward movement of the protein. This suggests that hydrolysis energy does not directly contribute to translocation (24,26). Instead, hydrolysis energy leads the protein to stabilize in its new position on ssRNA and return to its initial state for the next cycle. However, the rationale for the effect of hydrolysis energy is based on the conformational change of the protein in the product bound state. Consequently, we propose that hydrolysis alters the affinity of NS3 for products compared to substrates. This shift in affinity within the ATP-pocket creates a differential binding affinity within the RNA-cleft throughout the hydrolysis cycle. Therefore, investigating the NS3h structure while bound to both hydrolysis substrates and products is crucial for identifying the structural changes responsible for this differential nucleotide affinity at the ATP-pocket. These insights could be leveraged to develop strategies to inhibit NS3h function effectively.

Crystal structures of closely related WNV NS3h fail to elucidate the differential affinity of nucleotide (i.e. ADP) throughout the hydrolysis cycle. WNV NS3h shares sequence identity of 69% with its counterparts in DENV and ZIKV. Luo et al. (20) reported crystal structures of DENV NS3h+ssRNA complex, capturing states of the hydrolysis cycle. These structures reveal notable structural changes in residues coordinating the P_γ_ and P_i_ moieties, with Lys199 and Glu285 (Lys200 and Glu286 in WNV NS3h respectively) being closer to the P_γ_ moiety than the P_i_ moiety. In all these structures, P_β_ moiety of ATP or ADP is coordinated with Arg463 (Arg464 in WNV NS3h). It is crucial to emphasize that ADP must be released before the subsequent hydrolysis cycle can commence, implying a necessary alteration in ADP affinity which is a distinctive feature of motor proteins. However, the crystal structures of DENV alone do not provide insights into this aspect. A similar trend is observed in the crystal structures of ZIKV NS3h when bound to nucleotide and ssRNA, as reported by Yang et al. (27) and Tian *et al.* (28). Nevertheless, a comparison of these crystal structures reveals that ADP shifts outward more significantly in the post-hydrolysis-II state (ADP bound to NS3h+ssRNA) than in the intermediate state, while still interacting with Arg463 (Arg464 in WNV NS3h). Pérez-Villa *et al.* also reported differential affinity for ATP and ADP in their microsecond-long simulations of HCV-NS3h bound to substrates (29). This suggests the possibility of another low-affinity state for ADP, which remains unresolved due to the limitations of crystallization techniques for these viral helicases.

Sequence-wise distant to WNV, the tick-borne encephalitis virus (TBEV) NS3h crystal structures underscore the differential affinity based functional mechanism (30). As compared to DENV and ZIKV, contrasting structural change is noticed for TBEV NS3h Arg463 (Arg461 for WNV), which switches its direct contact with the P_β_ moiety in the presence of ATP or ADP+P_i_ to water-mediated contact in the ADP-bound TBEV NS3h. Interestingly, TBEV NS3h Arg466 (Arg464 for WNV) becomes detached from ADP in ADP bound NS3h of TBEV. These observations support the existence of differential nucleotide affinity as a functional mechanism of the NS3 helicase. However, the WNV NS3h shares 49% sequence identity with TBEV, which is lower than that of DENV or ZIKV. Of note, TBEV crystal structures are also unable to depict altered affinity for ADP in the presence of ATP and ADP+P_i_.

We hypothesize that the **motif-VI loop** plays a significant role in modulating the affinity for nucleotide at the ATP-pocket along the hydrolysis states. Arg464 and Arg461 are the arginine fingers within **motif-VI** responsible for stabilizing the nucleotide at the ATP-pocket. The structure of **motif-VI** comprises a very short stretch of α-helix 11 (α11) and a larger contiguous loop region that connects α-helix 11 and β-strand 14 (β14). To emphasize the loop structure, we have renamed **motif-VI** as **motif-VI loop (MVIL)**. While Arg461 is located in the helix region, Arg464 is situated in the loop region. Biochemical studies report the inhibitory potential of the motif-VI peptide as it is involved in ATP binding, and mutational studies have suggested a conformation-dependent loop function (31). In substrate-gated enzymes, the loop structures surrounding the active site have a significant effect, from substrate recognition to catalysis (32). Studies demonstrate that loop structures are not merely ornamental connectors of structured regions of proteins; instead, inherent conformational flexibility regulates protein functions, as evidenced in cases such as TPI (33), PTPs (34), BRD4-1 (35), etc. In some cases, enzyme activation becomes kinetically slow due to the need to attain the correct conformation of the loop (34) and strongly dictates the enthalpic contribution to protein-ligand binding affinity (35). Taken together, this evidence suggests the need to investigate the nucleotide-specific conformational dynamics of **MVIL**. Here, we report our examination of the differential affinity of NS3h-ADP, focusing on the influence of **MVIL** through theoretical modeling and extensive sampling of substrates bound to WNV NS3h and confirming our findings with experimental approaches.

## Materials and methods

### Model starting structures

In our study, we modeled substrates bound to WNV NS3h to identify the probable regulatory site of hydrolysis substrate binding and product release along the hydrolysis cycle. However, there are currently no substrates bound WNV NS3h structures available in the Protein Data Bank. WNV NS3h (KUNV) shares 68% sequence identity with DENV4. Therefore, we used DENV4 substrates bound structures (PDB ID: 2JLR, 2JLU, 2JLV, 2JLY and 2JLZ) as templates to generate ATP, ssRNA, ssRNA+ATP, ssRNA+ADP+P_*i*_ and ssRNA+ADP bound WNV NS3h structures through homology modeling using SWISS-MODEL (36). The ssRNA, ATP, ADP+P_i_, ADP and Mg^2+^ were extracted from the DENV4 structures and manually placed in their respective binding sites on the modeled WNV NS3h structure after alignment with the DENV4 structures. Further, the modeled ssRNA-bound WNV NS3h structure was used to prepare the D471E, D471N and D471L structures by changing only the terminal sidechain atoms of respective mutants.

### Simulation protocol

We conducted all-atom explicit water conventional molecular dynamics (cMD) simulations of the eight modeled structures, i.e. ATP, ssRNA, ssRNA+ATP, ssRNA+ADP+P_i_, and ssRNA+ADP-bound WNV NS3h protein along with three mutants (D471E, D471N and D471L) of ssRNA bound NS3h. We utilized the GPU-enabled AMBER18 software (37) to integrate the equations of motion, employing the ff14SB (38) and ff99bsc0χOL3 (39,40) force field parameters for the protein and ssRNA, respectively. Similarly, we used ATP (41), ADP (41) and Mg^2+^ (42) parameters, while P_i_ (H_2_PO_4_^−^) (23) parameters were adopted from our previous study. We retained the crystallographic water of DENV4 in our modeled structure to maintain the octahedral organization of Mg^2+^ surrounded by ‘O’ atoms. The neutral pH protonation state was assigned to amino acids based on the PropKa (43) generated p*K*_a_ values. Special attention was given to histidine residues for assigning protonation states, specifically HIE195, HID252, HID263, HIE268, HID274, HID288, HIE478, HIE488, HIE559 and HIE605. The protein complexes were solvated with TIP3P (44) water model in a cubic box, leaving a 20 Å buffer length between the protein and the box edge. Sodium (Na^+^) and chloride (Cl^−^) ions were added to neutralize the charge and maintain an ionic strength of 0.15 M in the solution using the ‘addIons2’ module of tleap. The resulting box volume was 107 Å^3^.

Each system was subsequently minimized in seven steps by gradually decreasing the restraint on the protein and substrates (including Mg^2+^) to maintain initial coordination, with each step running for 2000 steps of steepest descent. The restraint force constants used were 150, 100, 50, 10, 1 and 0.1 kcal ·mol^−1^ ·Å;^−2^ for steps one through six, respectively. Subsequently, we gradually heated the system from 0 K to 300 K over 500 ps in an NVT ensemble by applying a restraint of 150 kcal ·mol^−1^ ·Å^−2^ on the protein complex, followed by pressure (1 bar) equilibration for 1.4 ns in seven steps, gradually reducing the restraint force to relax the system. Finally, we performed a production run for 5 μs and 3 μs for wild-types and mutants respectively and generated conformations in an NPT ensemble. Periodic boundary conditions (PBC) were applied in all directions. The Langevin dynamics thermostat and Monte Carlo barostat were employed to maintain the systems at 300 K and 1 bar. Direct nonbonding interactions were calculated up to a 12 Å distance cutoff. The SHAKE algorithm was used to constrain covalent bonds involving hydrogen(45). The particle-mesh Ewald method was utilized to account for long-ranged electrostatic interactions (46). A 2 fs integration time step was applied, with energies and positions recorded every 10 ps. In total, we conducted approximately 34 μs of production simulations run.

### Cell lines

293T cells were acquired from ATCC and propagated in DMEM supplemented with 10% heat-inactivated FBS and 1% GlutaGro (Gibco). Cell lines were validated to be free of Mycoplasma contamination using a Mycostrip kit (Invivogen).

### Plasmids and generation of mutants

The lineage II WNV Replicon encoding GFP was a generous gift from Dr Ted Pierson at the NIH (47). To generate mutants, primers were ordered from Integrated DNA Technologies (IDT) encoding D → E, D → N or D → L mutations at position 471 in NS3. Site-directed mutagenesis was performed using InFusion cloning (Takara). The sequence of all replicon plasmids was validated using Oxford nanopore sequencing (Plasmidsaurus).

### Cell transfection and replicon assays

293T cells were seeded into 6-well plates and transfected with replicon plasmid using Xtreme-Gene9 transfection reagent (Sigma). 24 h post-transfection, cells were replated in duplicate 6-well plates for RNA or protein analysis and 96-well plates for automated immunofluorescence analysis. 48 hours post-transfection cells were collected in Tri-reagent (Zymo) or RIPA buffer supplemented with protease inhibitors and 96-well plates were fixed with 4% paraformaldehyde. For protease assays, cells transfected with an NS2b/3-Strep plasmid were collected in RIPA buffer supplemented with protease inhibitors 48 h post-transfection.

### cDNA generation and qRT-PCR

RNA was extracted from Tri-reagent using the Direct-Zol RNA extraction kit (Zymo). cDNA was generated using total RNA and M-MLV reverse transcriptase (Invitrogen). qPCR was performed to measure 18s rRNA and WNV replicon RNA against a standard curve of a pooled reference to calculate relative RNA abundance.

### Western blotting

Cell lysates were freeze-thawed to ensure complete lysis, then spun to remove cell debris. Clarified lysates were mixed with 6× Laemmli buffer and heated at 95^○^C for 5 min. 20 μg protein lysates were resolved on 10% tris/glycine polyacrylamide gels and transferred to PVDF (0.45 μm pore) before being blocked in 5% milk in TBST. Blots were incubated in primary antibodies overnight, then washed with TBST and incubated with HRP-conjugated secondary antibodies, washed, and developed using ECL (Cytiva).

### Immunofluorescence

After paraformaldehyde fixation, plates were washed 3× in PBS and incubated with Hoechst (5 μg/ml) for 1 h at RT. Plates were washed 3× in PBS and imaged using an ImageXpress Pico automated imaging system to quantify total cells and GFP expression.

### Protein expression cloning and purification

An *Escherichia coli* codon-optimized gene block encoding the helicase domain (aa171–619) of WNV NS3 was cloned into an IPTG-inducible, N-terminal 6xHis-tagging vector using In-fusion cloning to generate pET_6xHis-tev-coNS3h. Site-directed mutagenesis was performed to generate D471E, D471N, D471L and A287L mutations in the parental vector. Inducible vectors were transformed into competent Rosetta2 BL21-DE3 cells (Novagen) and grown in Terrific Broth containing ampicillin and chloramphenicol to an OD_600_ of 0.8, then expression of WNV NS3h was induced with 0.4 mM IPTG for 18 h at 14^○^C. Cultures were pelleted by centrifuging at 4^○^C and stored at −80^○^C. Fractions were collected pre- and post-induction to verify protein induction. Pellets were resuspended in IMAC lysis buffer (20 mM NaH_2_PO_4_, 300 mM NaCl, 10 mM imidazole, 5% glycerol pH 7.5) with one protease inhibitor tablet per 50 ml (Pierce). Pellets were lysed by mechanical disruption 10×, then spun at 3220×g for 60 min at 4^○^C to clarify the lysate. Lysates were loaded onto 0.5 ml packed bed of HisPur Ni-NTA resin (Pierce) and allowed to empty by gravity flow. Beds were washed with 20 bed volumes of IMAC wash buffer 1 (20 mM NaH_2_PO_4_, 300 mM NaCl, 25 mM imidazole pH 7.5) and wash buffer 2 (20 mM NaH_2_PO_4_, 300 mM NaCl, 90 mM imidazole pH 7.5), then eluted in 10 bed volumes of elution buffer (20 mM NaH_2_PO_4_, 300 mM NaCl, 250 mM imidazole pH 7.5). Fractions were collected for input, flow-through, wash 1, wash 2, and 1 bed-volume fractions were collected for the elution step. Peak elution fractions were pooled together and dialyzed against 1.25× storage buffer (500 mM NaCl, 62.5 mM HEPES pH 8.0) overnight at 4^○^C using Slide-A-Lyzer dialysis cassettes (10k MWCO). After dialysis, purified protein was quantified by BCA assay (ThermoFisher). Glycerol was added to a final concentration of 20% and protein was frozen in single-use aliquots and stored at −80^○^C (final buffer content 400 mM NaCl, 50 mM HEPES pH 8.0, 20% glycerol). Protein purity was calculated to be 93%.

### *in vitro* ATPase assay

1.5 ml reactions were assembled on ice as follows: 25 mM MOPS pH 6.5, 5 mg/ml BSA, 1.25 mM MgCl_2_, 1.5 mg/ml polyU RNA (Sigma), 0.01% Tween20, and 10 nM NS3h. ATP was added to a final concentration of 10 to 600 μM to start the reaction. Samples were incubated at 37°C and 100 μl samples were transferred at the indicated timepoints to a 96-well plate containing 10 μl 0.5 M EDTA on ice to stop the reaction. 75μl from each well was transferred to a fresh plate and incubated with 150 μl BioMol green (Enzo) to quantify inorganic phosphate from ATP hydrolysis. Product accumulation curves were plotted using Excel and the linear portion of each curve was used to calculate *V*_0_. Data were fit to a nonlinear regression and kinetic parameters were calculated using Graphpad Prism 10.

### Analyses

#### Conformation clustering

In this study, we employed the Size-and-Shape Space Gaussian Mixture Model (Shape-GMM) (48) to identify structural states (or clusters) of the **MVIL**. This model determines optimal parameters for multivariate Gaussians using particle positions as features. To determine the appropriate number of clusters, we utilized the elbow heuristic coupled with cross-validation (CV). The elbow heuristic suggests choosing a number of clusters at which point the log-likelihood as a function of number of clusters does not increase significantly. This is best quantified by the minimum in the second derivative of log-likelihood as a function of number of clusters. CV is used to ensure that the model has not been overfit. Five training sets were determine for sampling error. The assignment of a frame to a cluster was achieved by minimizing the Mahalanobis distance after a weighted (kronecker) alignment. The kronecker product model of the covariances was used in all shapeGMM analyses.

#### Linear discriminant analysis (LDA)

LDA is a dimensionality reduction algorithm for clusters separation that works by minimizing intra-cluster variance while maximizing inter-cluster variances. LDA on shapeGMM derived clusters has been found to produce good estimates of reaction coordinates separating the clusters (49). This supervised technique utilizes globally aligned and cluster-labeled particle positions to learn the cluster-separating vectors. LDA generates K-1 vectors that effectively best separate the clusters where K denotes number of clusters identified from ShapeGMM. We employed the Python library scikit-learn with the single-value decomposition (SVD) method to handle co-variance.

#### Binding enthalpy

In our NPT simulation, the binding enthalpy of the system is determined as the interaction energy between substrate (i.e. ADP) and protein. We consider the non-bonded interactions, including electrostatic and van der Waals interactions. We used Cpptraj *lie* module to calculate the binding enthalpy with a long-range cutoff of 12 Å.

#### Error analysis

The error of every presented metric from MD simulation data represents the standard deviation of means. To remove the time-correlation effect, the trajectory frames were randomly shuffled and chunked into several parts. The standard deviation is computed from the mean values of all chunks. For consistency between the clusters, each chunk contains 10^4^ frames.

#### Experimental statistical analysis

Statistical analyses were performed using GraphPad Prism 10. One-way ANOVA were performed with Dunnett’s correction for multiple comparisons.

### Model corroboration

Closest and conserved contact analysis of Mg^2+^, hydrolysis substrates, and ssRNA demonstrates well-modeled NS3h bound substrates and stable simulations. The Mg^2+^ ions form octahedral coordination with oxygen atoms. We computed Mg^2+^-coordinating oxygen atoms within 2.5 Å, and the results are tabulated in Supplementary Table S1. We observed that the octahedral arrangement is maintained throughout the simulation for all hydrolysis states. The Mg^2+^-T201 coordination is present in all hydrolysis states, while E286 is only part of the coordination shell in the ATP bound state. The participation of ATP or ADP oxygens in the coordination shell of Mg^2+^ decreases from the ATP-bound state to the ADP-bound state. Similarly, the Mg^2+^-surrounding water molecules increase from the ATP-bound to the ADP-bound NS3h. We also calculated the occurrence probability of conserved protein residues in the ATP-pocket (Supplementary Table S2) and RNA-cleft (Supplementary Table S3) that form contacts with ATP / ADP+P_i_/ ADP and ssRNA, respectively (see the table). Most of the contacts show a 99% existence probability, with only a very few contacts at 70%. However, the probability of contacts varies significantly between the hydrolysis states.

## Results and discussion

We present and discuss our in-depth analysis of the differential enthalpic affinity of WNV NS3 helicase for nucleotide hydrolysis, a critical factor in the hydrolysis turnover process that is essential for motor protein function. ATP hydrolysis involves a multistage process characterized by the changing identity of the nucleotide at the ATP-pocket. Based on available crystal structures of substrate bound NS3h, we can divide the hydrolysis cycle into four stable states (20,27,28,30). For the sake of clarity, we will refer to these states as hydrolysis states. These states include the apo state, where the ATP-pocket is vacant and only ssRNA is present at the RNA-Cleft of NS3h (ssRNA); the pre-hydrolysis state, where ATP enters the ATP-pocket of NS3h, transitioning it into the catalytic active form (ssRNA+ATP); the post-hydrolysis-I state, where NS3h is bound to the products (ADP and P_i_) of ATP hydrolysis (ssRNA+ADP+P_i_); and finally, the post-hydrolysis-II state, where NS3h is bound with ADP (ssRNA+ADP). After ADP release, NS3h returns to the apo state in preparation for the next cycle. Since ADP is a common nucleotide among the hydrolysis states, we present our analysis of nucleotide-protein affinity focusing on ADP (in case of ATP, we do not consider P_γ_ moiety). For clarity, we organize our results into five sections. In the first section, we report the predominant role of **MVIL** in altering the affinity of NS3h-ADP for hydrolysis states. The second section describes the conformational plasticity of **MVIL** and nucleotide-dependent sampling of **MVIL**. In the third section, we delve into a more detailed investigation of substrate-specific **MVIL** conformations and their regulatory role in nucleotide entry and exit. In the fourth section, we elucidate the inhibition of ATPase activity and delineate the molecular mechanism underlying a mutation at D471 of **MVIL**, distant from the active site. The last section describes results of D471 mutants on viral RNA replication.

### Motif-VI loop plays a predominant role in ATP-dependent differential binding enthalpy of NS3h-ADP

Experimental reports have been unable to elucidate the differential affinity of ADP in each hydrolysis state. Structural, biochemical, and mutagenesis studies suggest that motifs **I**, **II**, **III** and **VI** are involved in the ATPase activity of NS3h (10). Crystallographic structures have reported ssRNA induced structural changes in motif **I** and alterations in coordination, mostly in the P_γ_ moiety (20). However, limited attention has been given to the changes in ADP affinity. The crystallographic structures of DENV and ZIKV have failed to provide differential coordination for ADP in various hydrolysis states (20,28). While the structures of TBEV shed light on motif **VI** mediated ADP coordination changes in the post-hydrolysis-II state, they also fall short in explaining affinity changes in the post-hydrolysis-I state compared to the pre-hydrolysis state (30). These crystallographic reports are based on single structures bound to nucleotide and lack insights from conformational ensembles.

**Figure 2.**
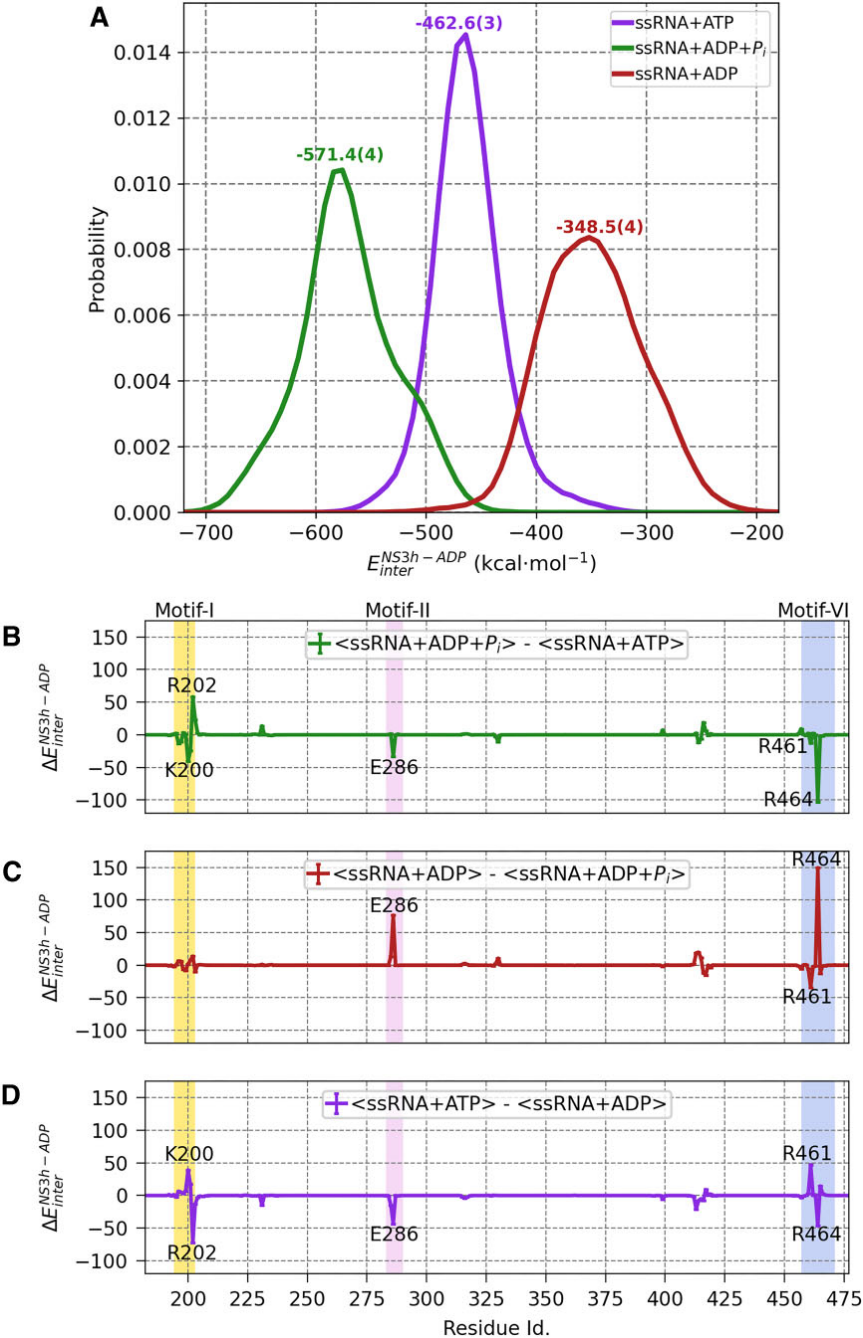
Nucleotide-dependent alteration of binding enthalpy of WNV NS3h and ADP along the hydrolysis cycle. (**A**) The binding enthalpy correspond to non-bonded linear interaction energy (E_inter_ in kcal· mol^−1^) of NS3h and ADP and here, presented as a probability distribution for ssRNA+ATP (pre-hydrolysis), ssRNA+ADP+P_i_ (post-hydrolysis-I) and ssRNA+ADP (post-hydrolysis-II) states. For the ssRNA+ATP system, we excluded the P_γ_ moiety in the calculation. The interaction energy calculation is performed over the entire 5μs of production run for each simulated system with *lie* module of *cpptraj*. Further, normalized histogram is calculated for 100 bins with same lower and upper bound in each system using python (*numpy.histogram*). In the figure, the mean value of each distribution is annotated by respective color with standard error of mean for last digit in parentheses. The difference of NS3h-ADP binding enthalpy between the states decomposed into protein residues which are presented as differential binding enthalpy for (**B**) ssRNA+ADP+P_i_ (post-hydrolysis-I) and ssRNA+ATP (pre-hydrolysis), (**C**) ssRNA+ADP (post-hydrolysis-II) and ssRNA+ADP+P_i_ (post-hydrolysis-I), (**D**) ssRNA+ATP (pre-hydrolysis) and ssRNA+ADP (post-hydrolysis-II). The ΔE_inter_ of a residue is computed by subtracting the mean of interaction energy of residue and ADP of corresponding substrates bound helicase (see Supplementary Figure S1). The error is propagated by following addition rule and presented as error-bar. *Matplotlib* is used to generate the plot.

WNV NS3h alters its enthalpic affinity for ADP according to the hydrolysis state as a functional mechanism of ATP-hydrolysis turnover. In Figure 2A, we present the distribution of interaction energy of the entire NS3h protein and ADP ($E^{NS3h-ADP}_{inter}$) for ssRNA+ATP, ssRNA+ADP+P_i_, and ssRNA+ADP systems with annotated mean values of −462.6(3), −571.4(4) and −348.5(4) kcal ·mol^−1^ respectively. This interaction energy can approximate the binding enthalpy, disregarding the pressure-volume terms and change in internal energy of NS3h and ADP. These distributions are statistically distinct, as quantified by a *t*-test (Supplementary Table S4). Compared to the pre-hydrolysis state, the binding enthalpy distribution of the post-hydrolysis-I state is left-shifted, indicating a stronger affinity. The post-hydrolysis-II state binding enthalpy is right-shifted compared to other states, indicating a weaker affinity of NS3h for ADP. We also computed binding enthalpy between NS3h and ADP+Mg^2+^, as this is the leaving group and Mg^2+^ stabilizes the products (see Supplementary Table S5). The values in the pre-hydrolysis, post-hydrolysis-I and post-hydrolysis-II state is −391.1(2), −461.9(3) and −350.8(8) kcal· mol^−1^ respectively. The change of NS3h and ADP+Mg^2+^ affinity along the hydrolysis states also coincides with the binding enthalpy of NS3h-ADP, while the former has lower magnitude of the affinity. These data suggest that after ATP binding, the protein favors hydrolysis, followed by favoring ATP binding for the next cycle after the release of products. We also note a hydrolysis state dependent differential interaction affinity between the protein and ssRNA (Supplementary Table S5). In the apo state, our computed interaction strength between WNV NS3h and ssRNA is −552.1(4) kcal ·mol^−1^. In the pre-hydrolysis, post-hydrolysis-I, and post-hydrolysis-II states, the protein–ssRNA interaction energies are −672.7(5), −746.8(6) and −647.6(7) kcal ·mol^−1^, respectively, which is stronger than in the apo state. Interestingly, the shifting trend of protein–ssRNA interactions in the hydrolysis states is similar to that of protein–ADP affinity and marked the functional importance of NS3h-ADP differential affinity at the active site. Moreover, we computed the MM-PBSA derived binding free energy (Supplementary Table S6) and its direction of change is consistent with NS3h-ADP binding enthalpy.

WNV NS3h provides enthalpic incentive for ATP hydrolysis as evidenced by the largest NS3h-ADP binding affinity in the post-hydrolysis-I state. Furthermore, the hydrolysis of the ATP substrate induces conformational changes in the protein, strengthening its grip on ADP. The strongest affinity for ADP in the post-hydrolysis-I also suggests that ADP is unable to release in this state. Transitioning to the post-hydrolysis-II state, with the weakest affinity, indicates that the protein has a weaker hold on ADP, enhancing its release. The enthalpic energy difference of 222.9(5) kcal ·mol^−1^ between the post-hydrolysis-II and post-hydrolysis-I state suggests that the presence of *P_i_* impedes the protein’s transition from the post-hydrolysis-I to the post-hydrolysis-II state enthalpically. Interestingly, the affinity between ADP and P_i_ in the post-hydrolysis-I state is highly repulsive (150.8(1) kcal ·mol^−1^). We hypothesize that the repulsive affinity between P_i_ with ADP facilitates P_i_ release followed by transition into post-hydrolysis-II state, a process often recognized as a rate-limiting step in motor proteins, as suggested in the literature (50–52). After ADP is released, it resets the protein to the initial apo state, allowing NS3h to bind the ADP moiety of ATP more strongly in the pre-hydrolysis state for the subsequent cycle than in the post-hydrolysis-II state of the previous cycle. It is important to note that the stronger affinity of NS3h for ADP in the pre-hydrolysis and post-hydrolysis-I states also reflects the influence of P_γ_ and P_i_, respectively. Nevertheless, the calculation of NS3h and ADP binding enthalpy has facilitated the comparison of these intriguing insights across all hydrolysis states.

**Motif**-**VI** **loop** (**MVIL**) emerges as a central player orchestrating the modulation of binding enthalpy between NS3h and ADP throughout the hydrolysis cycle. As illustrated in Figure 2B–D, we delve into the differential binding enthalpy of protein residues by subtracting the binding enthalpy of protein residues at different hydrolysis states, as illustrated in Supplementary Figure S1. Progressing through the hydrolysis cycle, we explore three transitions: the upper panel represents changes from the pre-hydrolysis state to the post-hydrolysis-I state, the middle panel delves into shifts from the post-hydrolysis-I state to the post-hydrolysis-II state, and the lower panel examines the transitions from the previous post-hydrolysis-II state to the next pre-hydrolysis state. In this analysis, K200, R202, E286, R461 and R464 exhibit significant deviation in their affinity for ADP among the hydrolysis states.

K200 and R202, situated within **motif-I** (P-loop), exhibit interesting motions. When the protein shifts from the pre-hydrolysis state to the post-hydrolysis-I state (Figure 2(B)), K200 fosters an attractive affinity, while R202 exerts a less attractive influence on ADP. Conversely, during the transition from the post-hydrolysis-II state (previous hydrolysis cycle) to the pre-hydrolysis state of next cycle (Figure 2(D)), we observe a complete reversal in trends, both in direction and magnitude. This suggests that K200 plays a crucial role in hydrolysis, while R202 contributes to stabilizing ADP (or ATP) in the P-loop during the pre-hydrolysis state. However, intriguingly, no significant changes emerge in the post-hydrolysis-II state compared to the post-hydrolysis-I state (Figure 2(C)), implying that K200 and R202 play a lesser role during this phase of change.

E286 functions as a base in ATP hydrolysis and displays varying degrees of repulsive affinity with ADP among the hydrolysis states. It exhibits less repulsion when transitioning from pre-hydrolysis to post-hydrolysis-I (Figure 2B), switches to more repulsive in post-hydrolysis-II compared to post-hydrolysis-I (Figure 2C), and turns less repulsive again when moving from post-hydrolysis-II to pre-hydrolysis (Figure 2D). This shift is due to the position of E286 relative to ADP; it is distant when hydrolysis will take place, creating less repulsion. In the absence of P_i_, E286 moves closer in the post-hydrolysis state-II, leading to large repulsion.

Significantly, the observed trend in hydrolysis state-dependent binding enthalpy changes underscores the prominent roles of **MVIL** residues, R461 and R464, in altering the overall binding enthalpy. As the protein moves from pre-hydrolysis to post-hydrolysis-I state (Figure 2B), R464 strongly binds to ADP, resisting its release, while R461 shows minimal change due to its close coordination with P_γ_ or P_i_. Transitioning to the post-hydrolysis-II state, R464 exhibits a significant change, become much less attractive to ADP (Figure 2C). This change is likely driven by the absence or release of P_i_, preparing the protein for recycling by releasing ADP. In the absence of P_i_, R461 moves closer to ADP, creating an attractive interaction. During the next cycle, R461 becomes less attractive, while R464 becomes more attractive in the pre-hydrolysis state compared to the post-hydrolysis-II state (Figure 2D), stabilizing ADP (part of ATP), perhaps for efficient access of P_γ_ for catalysis.

R461 and R464 in the **MVIL** exhibit localized correlations. In Supplementary Figure S2 we plotted the correlation of R461 and R464 with other residues of the protein. Notably, R464 exhibits a remarkably strong correlation, particularly in the absence of nucleotide at the ATP-pocket of apo state and to smaller extent in the post-hydrolysis states. Conversely, the pre-hydrolysis state shows the least correlation. These correlations suggest the conformational adaptability of the **MVIL** in the presence of nucleotide, which may play a crucial role in modulating its affinity for ADP.

### Conformational induction of motif VI loop along the hydrolysis cycle

The conformational dynamics of active site loops play a crucial role in catalysis, impacting substrate-specific interactions and loop-mediated processes. These loops, previously viewed as structural elements, are now recognized as functional contributors, capable of various roles beyond mere structural connections. Their inherent flexibility allows them to perform diverse functions and, often, to act as allosteric modulators (53). Notably, the active site loop’s conformational variations (34,54) influence substrate selection, intermediate state stabilization, and product release. In some enzymes, the sampling of catalytically active conformations is a rate-limiting step due to the wide range of possible conformations, affecting the catalytic rate. This conformational flexibility also leads to sampling of catalytically inactive states, potential targets for disrupting function through subtle changes (34,55,56). Additionally, substrate presence can tune loop conformations throughout the catalysis cycle, transforming a flexible loop into a rigid one (57). This substrate-dependent conformational change can be explained thermodynamically by either a *selection* or *induction* mechanism, involving the sampling of existing protein conformations or unique conformations, respectively (58). Distinguishing between these mechanisms is challenging, as both can occur depending on substrate concentration. A deeper understanding of these processes holds promise for targeted interventions in ATP hydrolysis.

Identifying unique conformations of the **MVIL** loop within the ensemble is crucial for understanding its regulatory role in the differential affinity for ADP throughout the hydrolysis cycle. This loop surrounds the active site (ATP-pocket) and is located opposite to the P-loop. Evolutionarily, loop sequences are highly conserved (32). Multi-sequence and structural alignment of crystal structures of DENV4, ZIKV, TBEV and WNV NS3 helicase demonstrate significant conservation of WNV **MVIL** (residues 461 to 472) among *Orthoflavivirus* members along with other motifs (Supplementary Figure S3). The strict conservation of the **MVIL** conformation in the apo state of NS3h underscores its importance in ATP hydrolysis and motivates to inspect the possibility of conformational change in the hydrolysis states. Therefore, we categorize the explored conformational ensemble of the **MVIL** and identify conformations specific to each hydrolysis state.

**Figure 3.**
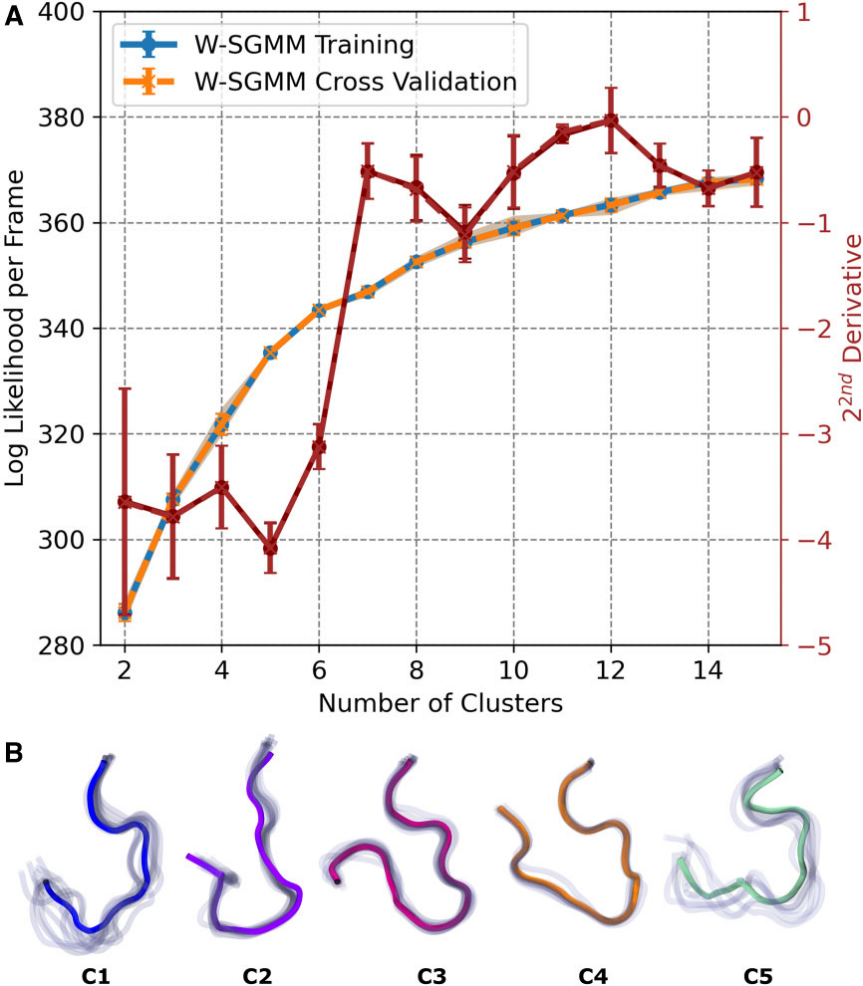
Clustering of motif-VI loop ensembles sampled in the hydrolysis states of WNV NS3h. (**A**) The log likelihood per frame as a function of clusters is plotted here with change of slope measure by 2^*nd*^ derivative. The clustering is performed with ShapeGMM algorithm by combining trajectories of simulated systems here. In our calculation, the feature consists with positions of all heavy atoms of residues 461 to 472. In this calculation, randomly 1 × 10^6^ frames are selected for training while prediction done on 1 × 10^6^ frames; each training run is iterated 5 times for each cluster. For change in slope, 2^*nd*^ derivative is averaged over all iterations of CV scan and error is calculated. (**B**) Structural representation of five **motif-VI loop** clusters based on the minima of slope change of log liklihood per frame. In each cluster, ten (10) randomly selected frames are displayed in translucent while the average structure is highlighted.

**Figure 4.**
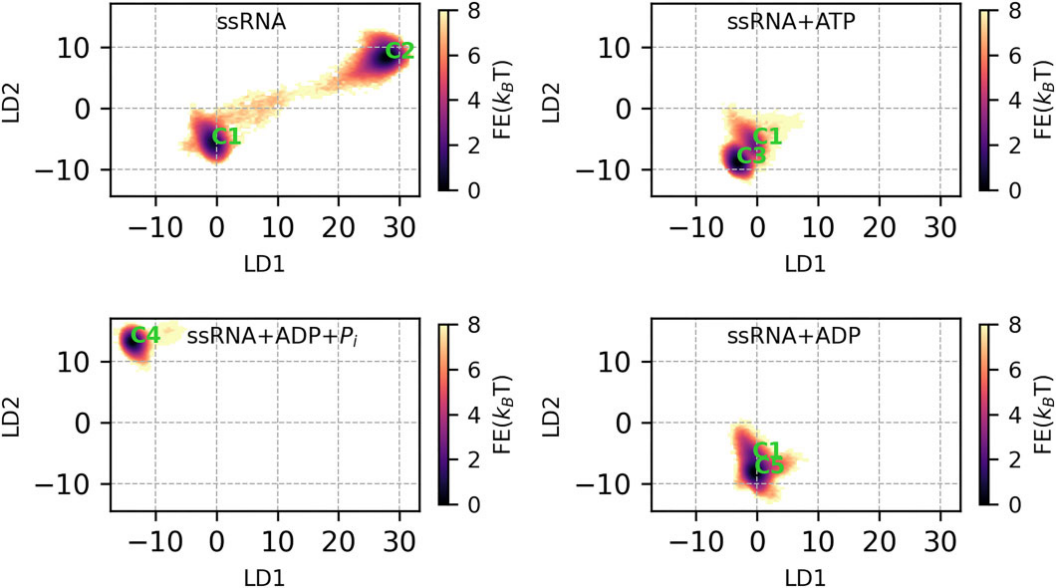
Free energy surface of **motif**-**VI** **loop** clusters in LD1 and LD2 space along the hydrolysis cycle. The free energy surface is presented over the LD1 and LD2 vectors computed by linear discriminant analysis (LDA) on the clustering identity tagged motif-VI loop conformational ensembles. Later, conformations belonging to ssRNA, ssRNA+ATP, ssRNA+ADP+P_i_ and ssRNA+ADP are projected along the LD1 and LD2 vectors followed by 2D histogram.

**Figure 5.**
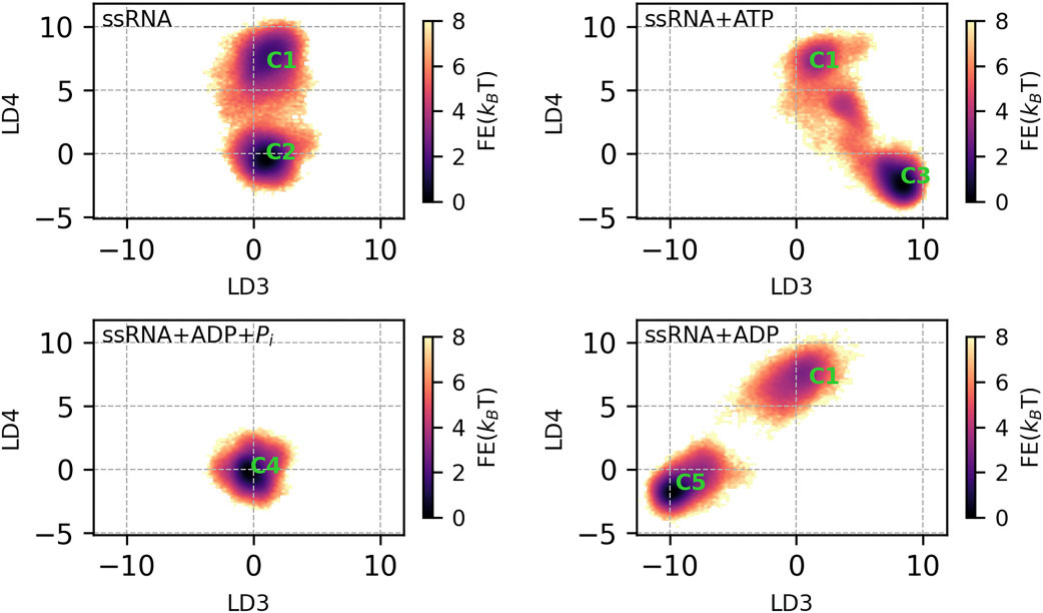
Free energy surface of **motif**-**VI** **loop** clusters in LD3 and LD4 space along the hydrolysis cycle. The free energy surface is presented over the LD3 and LD4 vectors computed by linear discriminant analysis (LDA) on the clustering identity tagged motif-VI loop conformational ensembles. Later, conformations belonging to ssRNA, ssRNA+ATP, ssRNA+ADP+P_i_ and ssRNA+ADP are projected along the LD3 and LD4 vectors followed by 2D histogram.

We identified five unique clusters (or states) within the conformational ensemble of the **motif-** **VI** **loop** using the weighted ShapeGMM (W-SGMM) clustering algorithm. In Figure 3A, we plotted the log likelihood per frame as a function of the number of clusters during a scan. This calculation was performed on the combined trajectories from apo (ssRNA), pre-hydrolysis (ssRNA+ATP), post-hydrolysis-I (ssRNA+ADP+P_*i*_), and post-hydrolysis-II (ssRNA+ADP) state simulations, totaling approximately 2 × 10^6^ frames. We focused on the heavy atoms of the **MVIL**. For the scan, we selected five training sets, each consisting of randomly chosen 1 × 10^6^ frames. The W-SGMM was fitted to the training set (blue curve), and cross-validation was performed on the remaining 1 × 10^6^ frames (orange curve). Both the W-SGMM training and cross-validation (red curve) exhibited a significant change in slope at five clusters, indicating the presence of five unique clusters sampled in the ensemble. After confirming the number of unique clusters, we clustered the entire combined trajectory into five, and representative structural images for each cluster are shown in Figure 3(B). To further demonstrate the uniqueness of these clusters, we projected the MVIL structures onto a 2D free energy surface using LD1–LD2 (Figure 4) and LD3–LD4 (Figure 5). The LD1 vector distinctly separated C1, C2 and C4 clusters, while LD2 separated C1 and C2. LD3–LD4 separation encompassed C1, C2, C3 and C5 clusters.

Nucleotide-dependent **MVIL** clusters suggest conformational induction at play during the hydrolysis progression. In Table 1, we present cluster probabilities for the apo (ssRNA), pre-hydrolysis (ssRNA+ATP), post-hydrolysis-I (ssRNA+ADP+P_i_), and post-hydrolysis-II (ssRNA+ADP) states. In the apo state, C1 and C2 clusters are sampled, with C2 being more prevalent (0.67) than C1 (0.33). Upon ATP binding in pre-hydrolysis state, the sampling of C1 is reduced (0.12), but C2 is not sampled. Instead, the pre-hydrolysis state predominantly samples the C3 (0.88) cluster of the **MVIL**. After hydrolysis, in the post-hydrolysis-I state, we do not observe the presence of C1, C2 or C3 clusters, only C4 cluster (1.0) is sampled. In the post-hydrolysis-II state, we notice the resampling of the C1 cluster with smaller probability (0.16), but C5 is the most frequently sampled cluster (0.84).

**Table 1.** ATP-dependent Motif-VI loop cluster probability

Clusters	ssRNA	ssRNA+ATP	ssRNA+ADP+P_i_	ssRNA+ADP
**C1**	0.33	0.12	–	0.16
**C2**	**0.67**	–	–	–
**C3**	–	**0.88**	–	–
**C4**	–	–	**1.0**	–
**C5**	–	–	–	**0.84**

These clusters are identified by shape-GMM clustering. Along the hydrolysis cycle, the presence or absence of hydrolysis substrate and products induced MVIL to predominately sample unique cluster.

It is important to note that each hydrolysis state displays the presence of a unique **MVIL** cluster. Our understanding of this differential sampling in line with the hydrolysis cycle is as follows: in the pre-hydrolysis state, the C1 cluster is selected for stabilizing the ATP binding at the ATP-pocket, and the binding induces C3 to provide a strong grip on ADP. We hypothesize that C3 represents the catalytic active state. Transitioning to the post-hydrolysis-I state, the presence of products induces the sampling of C4, which forms the strongest grip on ADP, preventing its release. After the release of P_i_, it induces the sampling of C5 in the post-hydrolysis-II state to weaken the grip on ADP, along with selecting the C1 cluster. This suggests that the preparation for the next hydrolysis cycle begins before the end of the previous cycle. ADP release induces C2 sampling, and in the absence of any nucleotide at the ATP-pocket, it leads to the sampling of C1 to prepare for the next hydrolysis cycle.

### Motif-VI loop acts as a valve for the nucleotide

**Figure 6.**
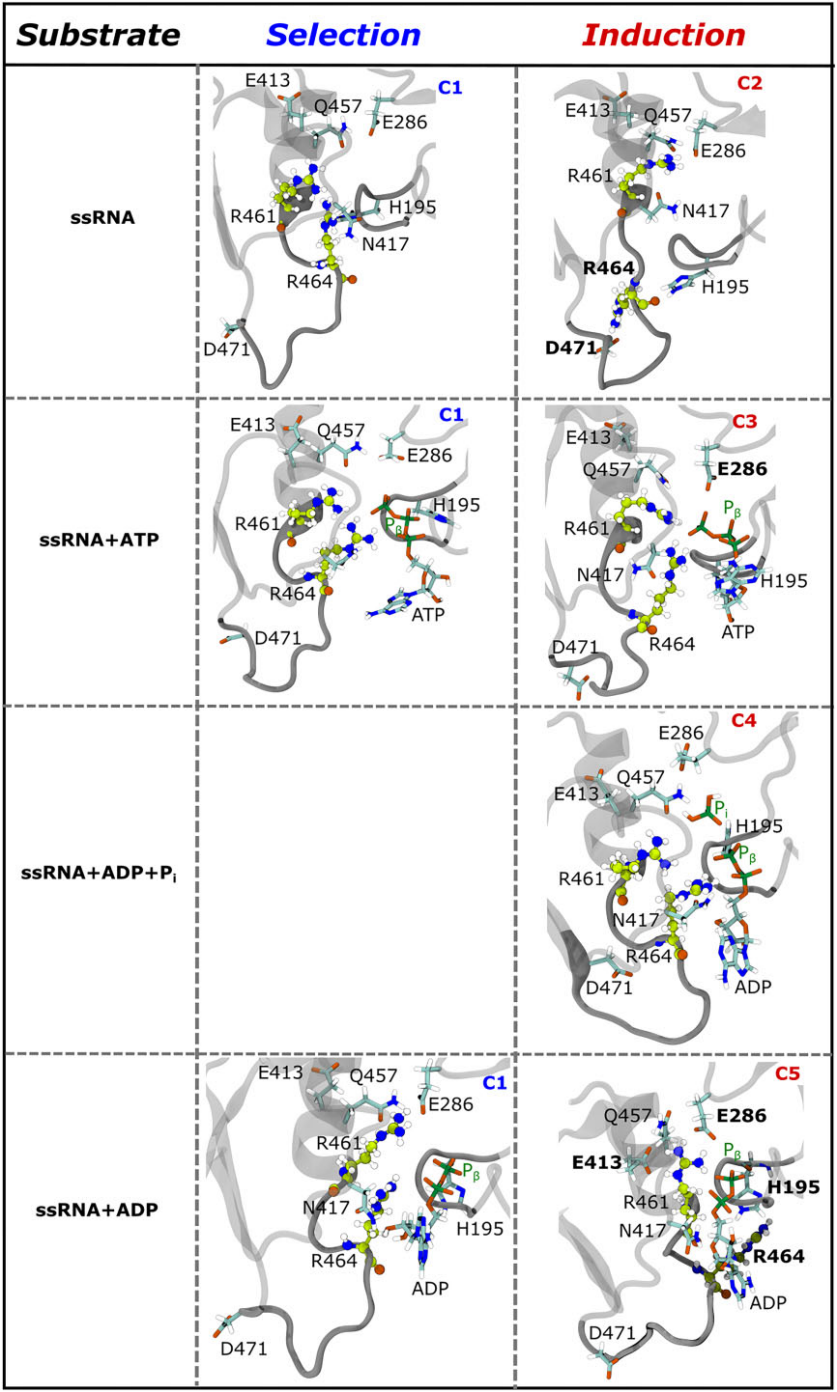
Nucleotide-dependent molecular depiction of conformational *selection* or *induction* mechanism of MVIL. From row 1 to row 4, nucleotide dependent **MVIL** structures are presented for ssRNA (apo), ssRNA+ATP (pre-hydrolysis), ssRNA+ADP+P_i_ (post-hydrolyis-I) and ssRNA+ADP (post-hydrolyis-II) states respectively. Structural representation **MVIL** clusters explored in each substrate system. R461 and R464 colored in green while closest contacts of these residues are presented in cyan.

In this section, we delve into the molecular mechanisms underlying NS3h-ADP affinity, particularly focusing on the key residues R461 and R464 within the **MVIL**. Our approach involves categorizing protein conformations based on **MVIL** cluster identity, hydrolysis state dependent separation, aligning them with the respective cluster means and co-variances of **MVIL**, and assessing the distances to the closest neighboring residues of R461 and R464 within a 5 Å; radius. For clarity, we present our findings separately for each hydrolysis state and provide visual representations of MVIL clusters and their neighboring residues in Figure 6. Detailed distance values for R461 and R464 are available in Tables 2 and 3, respectively, facilitating a comprehensive understanding of the molecular interactions involved.

**Table 2.** ATP-dependent R461(NH1) formed contacts distance and its deviation within the clusters

	ssRNA		ssRNA+ATP		ssRNA+ADP+P_i_	ssRNA+ADP	
Contact	C1	C2	C1	C3	C4	C1	C5
E286(OE1)	9.48(1)	4.77(1)	8.28(1)	6.18(1)	10.52(2)	2.94(1)	3.05(1)
E413(OE1)	10.97(2)	9.11(1)	10.13(1)	10.42(1)	10.23(1)	9.70(1)	6.50(2)
N417(OD1)	6.92(1)	4.21(1)	4.71(1)	6.23(1)	5.37(1)	8.40(1)	10.28(2)
Q457(OE1)	8.05(1)	4.78(1)	5.47(1)	6.13(1)	5.29(1)	4.73(1)	5.87(1)
P_γ_	–	–	3.98(1)	3.38(1)	–	–	–
P_i_	–	–	–	–	7.25(1)	–	–
P_β_	–	–	6.40(1)	6.02(1)	6.19(1)	5.20(1)	4.59(1)

Presented values denote average of the cluster in the respective hydrolysis state while parentheses value denote standard deviation of means of trajectory chunks for last digit. Units in Å. See error analysis for more details.

**Table 3.** ATP-dependent R464(NH2) formed contacts distance and its deviation within the clusters

	ssRNA		ssRNA+ATP		ssRNA+ADP+P_i_	ssRNA+ADP	
Contact	C1	C2	C1	C3	C4	C1	C5
H195(ND1)	7.09(2)	13.02(2)	10.62(1)	10.47(1)	11.55(1)	5.95(1)	4.48(1)
N417(OD1)	7.02(2)	17.93(2)	5.23(1)	4.53(1)	5.09(1)	11.27(2)	14.73(3)
D471(OD1)	20.54(2)	4.24(1)	17.66(2)	18.98(1)	11.16(1)	16.61(2)	20.07(2)
P_γ_	–	–	3.79(1)	3.80(1)	–	–	–
P_i_	–	–	–	–	6.67(1)	–	–
P_β_	–	–	5.03(1)	5.05(1)	4.41(1)	9.52(1)	9.66(1)

Presented values denote average of the cluster in the respective hydrolysis state while parentheses value denote standard deviation of means of trajectory chunks for last digit. Units in Å. See error analysis for more details.

In the apo state (ssRNA bound NS3h), **MVIL** exhibits both ‘open’ and ‘closed’ conformations. In C1 (row 1, middle panel of Figure 6), the R461 and R464 side-chains are oriented inward, towards the ATP-pocket, and both residues are in close proximity to N417 (∼7 Å). Additionally, R464 is also near to H195 (∼7 Å). This inward orientation of R461 and R464 in C1 results in a ‘closed’ valve-like conformation of **MVIL** around the ATP-pocket and we hypothesize that this is a nucleotide stabilizing conformation at the ATP-pocket. Conversely, in C2 (row 1, right panel of Figure 6), R461 moves further inward towards the ATP-pocket, where it is surrounded by E286, N417 and Q457, with a separation distance of ∼4–5 Å. Notably, in C2, R464 undergoes an outward motion relative to the ATP-pocket, representing a significant change compared to C1. This outward movement causes R464 to shift from ∼21 Å; away (in C1) to ∼4 Å; (in C2) close to D471, forming a salt bridge between R464 and D471. These rearrangements make C2 as ‘open’ valve-like conformation. Our simulation started from C1 (‘closed’) conformations of **MVIL**; however, we noticed 37 transitions from ‘open’ to ‘closed’ in 5 μs of simulation. This suggests R464 can spontaneously transition from the ‘open’ to ‘closed’ conformation within the limit of thermal energy, but the equilibrium is shifted towards the ‘open’ conformation due to large sampling of C2. This can be interpreted as the functional mechanism of nucleotide binding at the ATP-pocket: while **MVIL** is fluctuating between ‘open’ and ‘closed’ conformations, the nucleotide will encounter the ‘open’ conformation more frequently which facilitates nucleotide entry into the ATP-pocket. Following this, the **MVIL** transitions from the ‘open’ to ‘closed’ conformation where R464 interacts with the nucleotide to lock it into the ATP-pocket.

In the pre-hydrolysis (ssRNA+ATP-bound NS3h) state, the **MVIL** adopts ‘closed’ valve-like conformations to trap ATP at the ATP-pocket through an induction mechanism. Here, we observed the sampling of **MVIL** clusters C1 and C3. As suggested in the apo state, the inward motion of R461 and R464 in C1 (as in row 2, middle panel of Figure 6) forms close proximity to ATP to stabilize the nucleotide at the ATP-pocket. In this state, R461 is closer to the P_γ_ (∼ 4 Å) than the P_β_ (∼ 6 Å) of ATP. Interestingly, R464, is closer to both the P_γ_ (∼3.8 Å) and P_β_ (∼5 Å) of ATP in contrast to R461. These subtle changes support the importance of C1 as a *selective* conformation for ATP stabilizer to NS3h, effectively acting as a ‘closed’ valve. However, the presence of ATP *induces* a structural rearrangement in C1, bringing E286, N417, and Q457 residues even closer ∼1–3 Å to R461 and R464 than in the apo state. Upon ATP binding, **MVIL** transitions to the C3 cluster (as in row 2, right panel of Figure 6), R461 moves closer to the P_γ_ (∼3 Å) than in C1, but R464 maintains the same spatial distance from ATP phosphates. This results in N417 and Q457 moving ∼1 Å further away from R461 than in C1, while E286 moves closer to R461, though not as close as in C2. We hypothesize that these rearrangements in C3 lead the ATP-pocket (i.e. active site) to transition to a catalysis-active conformation while **MVIL** remains as a ‘closed’ valve by stabilizing the ADP portion of ATP through contact with R464. We computed the probability of catalytic water oxygen (O_W_) within (i) 5 Å of the P_γ_ ATP atom and (ii) an angle of 150^○^ to 180^○^ between the vectors O_β, γ_-P_γ_ and P_γ_-O_W_. Davidson *et. al.* noted this geometry’s importance for catalysis (23). In our analysis, we found the probability of catalytic water to be 5.33% for C1 and 31.67% for C3. This significant difference supports that the C3 represents a catalysis active state. Moreover, we found that **MVIL** of NS3h bound ATP largely samples C1 (Supplementary Figure S4), demonstrating RNA-stimulated influence on **MVIL** conformation.

In the post-hydrolysis-I (ssRNA+ADP+P_i_-bound NS3h) state, conformational induction of **MVIL** resists ADP release. The presence of both hydrolysis products *induces* sampling of the C4 cluster of **MVIL** in the ssRNA+ADP+P_i_ bound NS3h. In C4 (as in row 3, right panel of Figure 6), R461 moves ∼ 7 Å further from P_i_ as compared to P_γ_ but maintains similar spatial separation from P_β_ as in C1 and C3 of the pre-hydrolysis state. This results in close proximity of R461 to N417 and Q457 than C3. Similarly, R464 is also more distant from P_i_ as compare to P_γ_. Interestingly, R464 moves ∼1 Å closer to P_β_ than in the pre-hydrolysis state. This results in the strongest affinity for ADP compared to the pre-hydrolysis state, thereby resisting ADP release.

In the post-hydrolysis-II (ssRNA+ADP-bound NS3h) state, **MVIL** adopts an ‘open’ valve-like conformation. This state, with only ADP present (or lacking P_i_), leads to sampling of C1 and C5 MVIL loop conformations. In C1 (row 4, middle panel of Figure 6), R461 becomes ∼1 Å closer to P_β_ than previous states. The motion of E413 and N417 surround the R461 is *selected* for C1 of apo state while closest proximity of E286 and Q457 to R461 is an *inductive* effect in the presence of (only) ADP. However, R464 moves ∼6 Å further away from P_β_ but inward towards the ATP-pocket and becomes ∼6 Å closer to H195 than in the post-hydrolysis-I state. In the C5 cluster, R461 becomes subtly closer to P_β_ (∼4.6 Å); more distant from N417 and Q457. In this cluster only, we observe R461 in close contact with E413 at ∼6 Å, as compared to the other clusters (∼10 Å). Most importantly, R464 separates from ADP, shifting away from the ADP plane (∼9 Å) or outward from the ATP-pocket than in C1 and becomes closest to H195 (∼4 Å). This opens the ATP-pocket, generating a repulsive effect on ADP and facilitating ADP release for the next hydrolysis cycle.

### Mutations at D471 probe the proposed valve behavior of MVIL

We propose D471 as a potential site for mutation to indirectly affect the ATP-pocket (i.e. active site) function. The R461 and R464 residues undergo critical inward motions during hydrolysis, causing the ATP-pocket to be ‘closed’, which is essential for ATP hydrolysis. Targeting these residues would disrupt the ATP hydrolysis function of WNV NS3h. However, this approach is common yet challenging for helicases due to specificity issues, as the active site motifs are widespread and conserved in host factors, potentially leading to detrimental effects. The outward orientation of R464 causing an ‘open’ ATP-pocket likely affects ATP stabilization in the ATP-pocket (C2) or facilitates ADP release (C5). Both conformations involve the interaction of R464 with D471 or H195. While H195 is unsuitable for disruption due to its location in the P-loop, D471 is a viable choice because it is distant from the active site and is solvent-exposed. This R464-D471 salt bridge mediated the **MVIL** ‘open’ valve-like conformation (C2), which is only sampled in the apo state along with the ‘closed’ valve-like conformation (C1). Our proposal involves substituting D471 to maintain either a similar sidechain chemical nature or sidechain length through substitutions like D471E, D471N and D471L. We hypothesize that these substitutions may favor any of the **MVIL** conformations observed in the wild-type apo state. This, in turn, could impact ATP hydrolysis followed by replication inhibition by either destabilizing (favoring ‘open’) or blocking (favoring ‘closed’) ATP binding.

**Figure 7.**
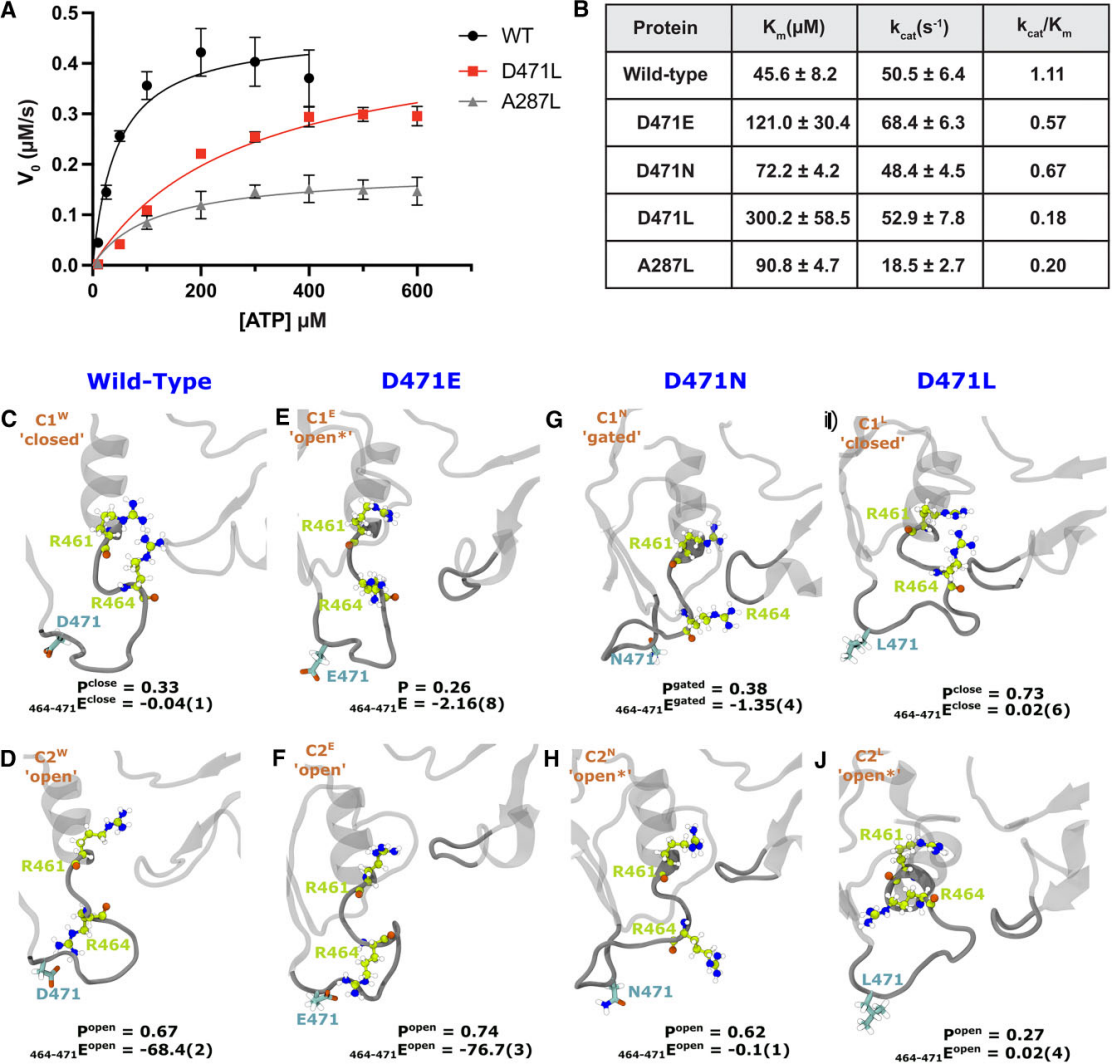
Mutations at D471 reduce ATPase activity by influencing MVIL conformations to affect ATP binding. The top panel denotes experimental results of ATPase assay. (**A**) A plot of V_0_ vs. ATP concentration for Wild-type, D471L, and A287L mutants in NS3h. Data are fit to a nonlinear regression from which K_m_ and k_cat_ were calculated. (**B**) Table of Michaelis–Menten kinetic parameters of wild-type and D471 mutant NS3h. Catalytic efficiency was calculated by dividing k_cat_ by K_m_. In the middle and lower panel, we present representative structures of **MVIL** clusters of wild-type (WT) and mutants identified by shapeGMM clustering: (**C**, **D**) WT, (**E**, **F**) D471E, **G**, **H**) D471N and (**I**, **J**) D471L. Marking of each cluster is based on the 461–464 and 464–471 side chain distances tabulated in Supplementary Table S7. In each structural illustration, the probability (P) and mean electrostatic interaction energy (_464−471_E in kcal ·mol^−1^) between 464–471 is annotated for its respective **MVIL** clusters. For _464−471_E, the standard deviation of means of last digit is presented in parentheses. See error analysis for standard deviation calculation.

To test this hypothesis, we engineered the WNV NS3h gene into an inducible *E. coli* vector for recombinant protein expression and purification. We first purified wild-type and D471 mutant NS3h from *E. coli*, quantified protein concentration using a BCA assay, and calculated purity by SDS-PAGE (Supplementary Figure S5(A)). We then used purified NS3h to perform an *in vitro* ATP hydrolysis assay and measured the rate of free phosphate accumulation at multiple ATP concentrations (Supplementary Figure S5(B)). We calculated V_o_ for each ATP concentration and fit these data to a nonlinear regression to determine the kinetic parameters for the recombinant protein (Figure 7(A)). We found that the wild-type NS3h has a K_m_ of 45.6 ± 8.2 μM and a k_cat_ of 50.5 ± 6.4 s^−1^ (Figure 7(B)). As a positive control for this assay, we used a recombinant NS3h with an alanine to leucine substitution at amino acid 287 (A287L), within the DEAH motif of NS3h, which has previously been shown to reduce the ATP hydrolysis activity of *Orthoflavivirus* NS3h (59). As expected, the A287L mutant NS3h had an increased K_m_ of 90.8 ± 4.7 μM and a decreased k_cat_ of 18.5 ± 2.7 s^−1^, with substantially reduced catalytic efficiency as compared to wild-type (20% of wild-type, Figure 7(B)). We also purified NS3h bearing D471E and D471N substitutions, which we hypothesized would have a more modest effect on ATP hydrolysis, since the interaction between D471 and R464 should remain intact. Indeed, we found that D471E NS3h had a K_m_ of 121.0 ± 30.4 μM and a k_cat_ of 68.4 ± 6.3 s^−1^, while D471N NS3h had a K_m_ of 72.2 ± 4.2 μM and a k_cat_ of 48.4 ± 4.5 s^−1^ (Figure 7(B)). Among the D471 mutant NS3h proteins, we found that the ATPase activity of the D471L mutant was the most markedly impaired, with a K_m_ of 300.2 ± 58.5 μM and a k_cat_ of 52.9 ± 7.8 *s*^−1^ (Figure 7(B)). These data are consistent with our hypothesis that the D471-R464 salt bridge is required for efficient ATP hydrolysis by the NS3h protein, since the nonpolar sidechain of the mutant L471 should be unable to interact with R464. The catalytic efficiencies (k_cat_/K_m_) of the D471E and D471N mutants were at 57% and 67%, respectively, while the D471L substitution reduced catalytic efficiency to 18% of wild-type activity (Figure 7B). Together, these data show the importance of the D471 residue and demonstrate that disruption of the D471-R464 salt bridge inhibits the ATP hydrolysis activity of recombinant NS3h protein in an *in vitro* ATPase assay.

To understand the mechanism of ATPase inhibition by the mutants, we simulated D471E, D471N, and D471L WNV NS3h bound to ssRNA (apo state) followed by identifying unique conformational clusters of MVIL (heavy atoms of residues 461 to 472) with weighted ShapeGMM (W-SGMM). The scan analyses show that each mutant explores two unique conformations of **MVIL** (see Supplementary Figure S6). Due to the differences in particles of **MVIL** between wild-type and mutants, we used side chain distances of R461–R464 and R464–D471 to mark the clusters as ‘open’ or ‘closed’ (see Supplementary Table S5). When R464 is inward and close to R461, we denote such cluster as ‘closed’, while the cluster in which R464 is outward but forming salt-bridge with D471 ‘open’. With this description, wild-type C1^W^ and C2^W^ marked as ‘closed’ and ‘open’ respectively with sampling probability of 0.33 and 0.67 respectively (Figure 7C, D).

The **MVIL** of D471E destabilizes ATP binding for hydrolysis. We chose D471E mutation as a subtle modification which can maintain the chemical nature of wild-type while extending the side chain. The **MVIL** explored two conformations C1^E^ and C2^E^ with sampling probability of 0.26 and 0.74 respectively (Figure 7E, F). We denote C1^E^ as an ‘open*’ conformation as R461-R464 are distant but R464 is not forming a salt bridge with E471, while *C*2^*E*^ as an ‘open’ conformation because R464–E471 form a salt bridge. Interestingly, the sampling probability of **MVIL** ‘open’ conformation is 0.74, which is higher than the wild-type. This indicates that D471E predominantly favors the ‘open’ ATP-pocket, allowing ATP to enter the active site. However, due to the strengthening of the R464–E471 salt bridge in the mutant (-76.7(3) kcal· mol^−1^) compared to R464–D471 in the wild-type (–68.4(2) kcal ·mol^−1^), R464 is restricted from switching inward and is unable to stabilize the ATP for hydrolysis. Hence, the ATP-binding efficiency is decreased (K_m_ is increased) in the D471E mutant compared to the wild-type.

Next, we explored the D471N mutation to evaluate the salt bridge effect by maintaining the structure but changing the chemical nature of the side chain from negatively charged to polar. In this mutant, the **MVIL** explored C1^N^ and *C*2^*N*^ clusters of conformations with sampling probability of 0.38 and 0.62, respectively (Figure 7H, I). In C1^N^, R464 is distant from both R461 and N471 but is closer to the P-loop such that it is guarding the ATP-pocket; we named this cluster as ‘gated’ which inhibits the entry of ATP to the active site. In contrast, R464 is distant from R461, N471 and also P-loop in the C2^N^ cluster. We designated C2^N^ as ‘open’ which facilitates ATP entry. The sampling of the C1^N^ conformation resulted in reduced (increased) ATP-binding (K_m_) efficiency in the D471N mutant. However, due to the smaller probability of the inhibitory conformation in D471N compared to D471E or D471L mutants, the ATP-binding efficiency is higher in D471N than in D471E or D471L mutants.

Mutating D471 to L blocks ATP entry to the active site. The motivation for the ‘D’ to ‘L’ substitution is to maintain the sidechain length but significantly change the chemical nature. However, D471L apo state only explored C1^L^ and C2^L^ clusters of **MVIL** with sampling probability of 0.73 and 0.27, respectively (Figure 7(I-J)). In C1^L^, R464 is inward and closer to R461 and, thus, we are referring to it as ‘closed’ conformation. In contrast, R464 is outward but unable to form contact with L471 and hence it is marked as ‘open*’ conformation. This suggests that D471L predominately favors the ‘closed’ conformation of **MVIL** in the apo-state and blocks ATP entry. This resulted in decreased ATP-binding efficiency in the D471L mutant as compared to the wild-type and other mutants, as demonstrated by largest increase of K_m_. All the modeled mutants showed sampling of an ATP-binding or -stabilizing competent **MVIL** conformation, which led to hydrolysis in our *in vitro* ATPase assay; however, each mutant displayed a departure from the ‘open’–‘closed’ sampling frequency and conformation compared to the wild-type, which resulted in the observed sub-optimal ATP hydrolysis activity. The combination of experimental kinetic data of ATPase and computational modelling of **MVIL** structural data suggest that the R464-D471 salt-bridge regulates the valve-like nature of **MVIL**.

### MVIL mutants impair WNV replication *in vitro*

**Figure 8.**
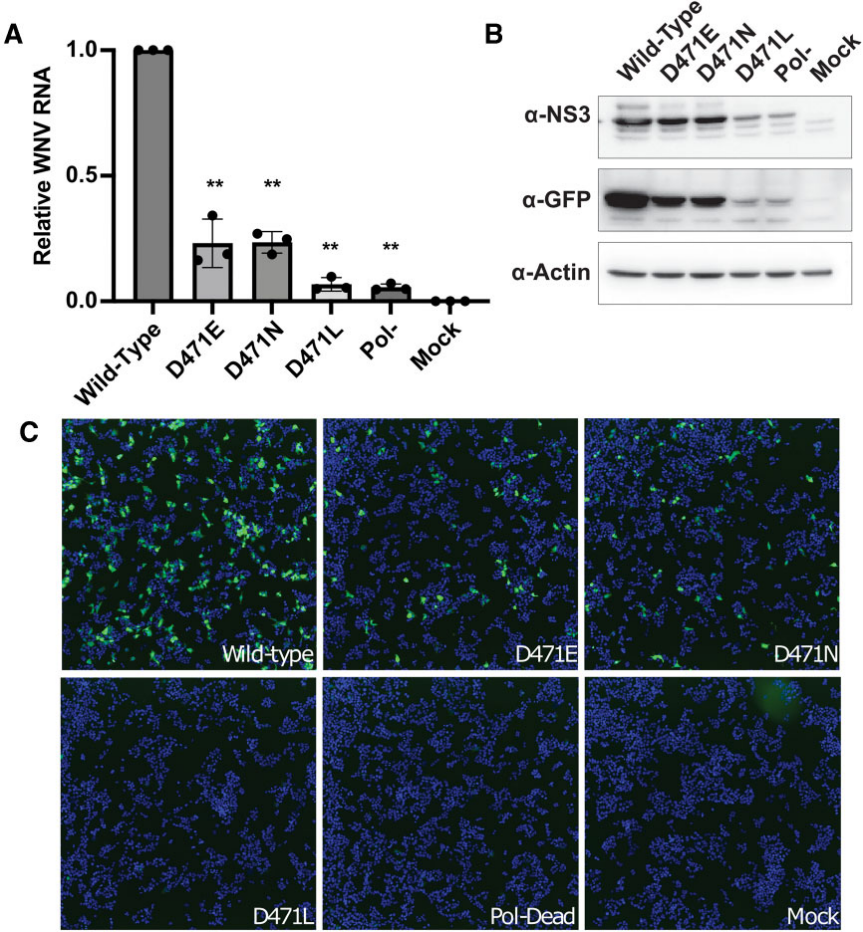
D471 substitutions inhibit WNV RNA replication. (**A**) qRT-PCR of cells transfected with the indicated wild-type or mutant WNV replicon. Data shown are normalized to wild-type, *n*= 3, statistics are one-way ANOVA with Dunnetts correction for multiple comparisons, performed on raw non-normalized data, ** *P*< 0.01. (**B**) Western blot of NS3 and GFP proteins encoded by the replicon transfected in (A). (**C**) Fluorescence microscopy of GFP and Hoechsts stain in replicon-transfected cells.

Our *in vitro* biochemical data and modeling suggest that disruption of the D471–R464 salt bridge may impact viral RNA replication by inhibiting ATP hydrolysis by NS3h. To test this hypothesis, we utilized a WNV subgenomic replicon. This subgenomic replicon is a subviral genome capable of autonomous viral RNA replication but harbors a 5′ deletion of the structural genes C, prM and E, which are replaced with a GFP reporter. This replicon cannot generate infectious virions, but offers a sensitive approach to measure the impact of amino acid substitutions at specific residues on viral RNA replication. We engineered replicons with substitutions at D471 (D471E, D471N, D471L), transfected them into cells, and performed qRT-PCR to assess impacts on viral RNA replication. We found that substitution of D471 with either E or N reduced RNA replication 4-fold (Figure 8(A)). However, substitution of D471 with L reduced RNA replication 14-fold, to a level similar to a replicon harboring a mutation in the NS5 RdRp catalytic site, D668A (Pol-dead, 8(B)). We confirmed these data by immunoblotting for the GFP reporter, as well as the mutant NS3, from cells transfected with mutant replicons (Figure 8(B)). Additionally, we used automated fluorescence microscopy and quantified the number of cells expressing the GFP reporter for each mutant replicon (Figure 8C, Supplementary Figure S7). These data agree with our *in vitro* biochemical data, where we observed a moderate reduction in catalytic efficiency for the D471E and D471N mutants (Figure 7B), while D471L had a steep decrease in catalytic efficiency compared to wild-type.

In the context of infectious virus, WNV NS3 has both protease and RNA helicase activities, both of which are required for RNA replication. Substitutions at NS3 D471 could theoretically impact protease activity of NS3, which could inhibit RNA replication independent of their influence on ATP hydrolysis. Therefore, to assess non-helicase related impacts of D471 substitutions, we performed a protease self-cleavage assay using a WNV NS2b/3 expression vector. Expression of NS3 with its cofactor, NS2b, as a polyprotein (NS2b/3) leads to self-cleavage and liberation of the NS2b protein, which can be measured by western blotting. We transfected this vector into cells and measured NS2b/3 self-cleavage into NS2b and NS3 by western blot (Supplementary Figure S7). We also engineered D471 substitutions into the NS2b/3 expression vector and found similar protease efficiency compared to wild-type (Supplementary Figure S7), while a known protease catalytic mutant, S135A, was unable to cleave NS2b from NS3 (Supplementary Figure S7). These data show that full-length wild type and mutant NS3 proteins are translated efficiently in mammalian cells, fold properly, and do not have impaired protease activity.

Taken together, our data show that the ATP hydrolysis activity of NS3h is highly dependent upon the ability of D471 to form a salt bridge and that disruption of this interaction, even with a highly conservative D471E substitution, significantly reduces the efficiency of ATP hydrolysis. These impacts on ATP hydrolysis significantly impair the ability of viral RNA to replicate, since the ATP-dependent RNA helicase activity of NS3 is required to unwind the dsRNA replication intermediate produced during viral replication.

## Conclusion

WNV NS3h demonstrates a differential binding enthalpy mechanism of WNV NS3h-ADP to allow for ATP turnover. To demonstrate this, we quantified the ADP-protein interaction energies from extensive all-atom explicit solvent MD of WNV NS3 in ssRNA, ssRNA+ATP, ssRNA+ADP+P_i_, and ssRNA+ADP-bound states. A favorable enthalpic change between the ssRNA+ADP+P_i_ (post-hydrolysis-I) and ssRNA+ATP (pre-hydrolysis) states indicates an additional enthalpic driving force for hydrolysis provided by NS3h. Interestingly, the unfavorable change in binding enthalpy between the ssRNA+ADP (post-hydrolysis-II) and ssRNA+ADP+P_i_ (post-hydrolysis-I) states suggests a dependence on the release of P_i_. Furthermore, evidence of the progression to the next hydrolysis cycle is also apparent through the favorable enthalpic change between the ssRNA (pre-hydrolysis) and ssRNA+ADP (post-hydrolysis-II) states. This differential binding enthalpy has signicifant contribution from R461 and R464 located in the **MVIL** residues. Nucleotide-specific motion of R463 and R466 (R461 and R464 in WNV, respectively) is also evident in the crystallographic report of TBEV (30). From Shape-GMM clustering analysis, we found that the differential binding affinity is regulated by **MVIL** conformations and demonstrated conformational induction at play along the hydrolysis cycle. Among the induced **MVIL** clusters, C2 and C5 represent as a ‘open’ valve-like structures whereas C3 and C4 resemble ‘closed’ valve-like structures. This difference originated from the R464 structure relative to the ATP-pocket. Among the ‘open’ structures, in C2, the outward structure of R464 leads to the formation of a new salt bridge with D471, distant from the active site. This is the first report of the possibility of an R464-D471 salt bridge, which results in an ‘open’ valve-like structure of **MVIL** in the apo state (NS3h bound to ssRNA). Moreover, D471 is a conserved residue within the *Orthoflavivirus* genus. Thus, it indicates this salt bridge formation could be a common structural feature and a potential site of indirect disruption of active site function.

Our clustering analyses of MVIL and the motion of R464 during ATP hydrolysis led us to investigate the role of the D471 residue on regulating ATP hydrolysis in NS3h. We used multiple strategies, including *in vitro* biochemical assays and viral replication assays, to assess the impacts of D471 substitutions on viral protein activity and viral replication. Our *in vitro* biochemical data show that ablating the D471-R464 salt bridge steeply reduces the efficiency of ATP hydrolysis, consistent with an important role for D471 in the regulation of NS3h ATPase activity. Modelling and simulation of these mutants in the apo state show sampling of two conformational clusters of **MVIL**. The D471E and D471L substitutions display shifts from the wild-type population of **MVIL**, predominantly sampling ‘open’ and ‘closed’ valve-like conformations, respectively. These observations suggest a probable mechanism of hydrolysis reduction: D471E is unable to stabilize ATP in the ATP-pocket after ATP binds in the ‘open’ conformation, while in D471L NS3h, ATP is unable to enter the ATP pocket due to a ‘closed’ ATP-pocket. Interestingly, in the D471N simulation we could not discern a probable mechanism of hydrolysis disruption, but observed sampling of closed-like conformations of **MVIL** to a smaller extent which can impact ATP entry. Further, our western blot and qRT-PCR results from the viral replication assay show that D471 mutants significantly reduce viral RNA replication. This is consistent with D471 regulating ATPase activity of WNV NS3. Indeed, though we were able to detect some catalytic activity of D471L NS3h, the greatly reduced catalytic efficiency of the protein resulted in limited D471L replicon RNA amplification, similar to our RNA-dependent RNA polymerase catalytic mutant control, D668A. Importantly, the results from our protease activity assay suggest that these D471 mutant proteins are expressed and properly folded, as they retain protease activity similar to the wild type construct.

Altogether, these evidences have showcased the differential affinity-based functional mechanism of NS3h hydrolysis turnover, which is regulated by the valve-like dynamics of the **MVIL** conformations. Analogous to a combustion engine, initially (apo state), MVIL exists as an open valve. Once the fuel (i.e. ATP) enters the chamber (i.e. ATP-pocket), **MVIL** transforms into a closed valve, locking the fuel (pre-hydrolysis and post-hydrolysis-I states). After combustion (i.e. ATP hydrolysis), the valve opens the chamber for the release of the product (i.e. ADP in post-hydrolysis-II state). However, this is not a simple mechanical process but is underlined by the complex dynamics of conformational induction. This study provides an in-depth understanding of the critical role of the **MVIL** in hydrolysis states in WNV NS3h, beyond its simple function as a nucleotide stabilizer. The **motif VI loop** is a surface-exposed structure and can be leveraged to target the hydrolysis state-dependent conformation to abolish its hydrolysis function, as observed in our D471 mutant NS3h, rather than directly targeting the ATP-pocket, which could be detrimental to host helicases. Structurally, NS3h shares a similar arrangement within the *Orthoflavivirus* genus and exhibits high sequence similarity (see Supplementary Figure S3). Therefore, we believe that these insights will aid in understanding the ‘valve’-like function of the **MVIL** region in other virus helicases within the *Orthoflavivirus* genus. In addition to catalysis, the active site loop possesses a triggering or triggered loop effect, as seen in the WPD loop (32). Future work in our group will explore such possibilities in the WNV NS3h helicase.

## Supplementary Material

gkae500_Supplemental_File

## Data Availability

The data underlying this article will be shared on reasonable request to the corresponding authors.
