# Supplementary material for: Motif-VI loop acts as a nucleotide valve in the West Nile Virus NS3 Helicase
Source: Nucleic Acids Res. 2024 Jun 17;52(13):7447–64. doi: 10.1093/nar/gkae500 (PMC11260461; doi:10.1093/nar/gkae500)

Supplementary data of

Motif-VI Loop Acts as a Nucleotide Valve in the West Nile Virus NS3 Helicase

Priti Roy†, Zachary Walter†, Lauren Berish, Holly Ramage* and Martin McCullagh*

Email: martin.mccullagh@okstate.edu; holly.ramage@jefferson.edu

1 Analyses

1.1 MM-PBSA

The MM-PBSA method is utilized to compute the binding free energy between the NS3h protein and ADP for the pre-hydrolysis, post-hydrolysis-I and post-hydrolysis-II states in implicit water condition with internal dielectric constant of 1. We used single-trajectory approach and thus, individual solvation part of both the ligand (i.e. ADP) and receptors (i.e. protein) is coming from the complex simulation in explicit water. For each state, we considered approx. 500 frames. To note, this binding free energy excluded of the entropic change of protein-ligand binding.

1.2 Primers

**Primer Name**	**Sequence 5′-3′**	**Purpose**
Rep-D471E-F	AGTTGGTGAGGAGTATTGCTATGGAGGGCACAC	Mutagenesis
Rep-D471E-R	TACTCCTCACCAACTTGTGATGGGTTTCTTCC	Mutagenesis
Rep-D471N-F	AGTTGGTAACGAGTATTGCTATGGAGGGCACAC	Mutagenesis
Rep-D471N-R	TACTCGTTACCAACTTGTGATGGGTTTCTTCC	Mutagenesis
Rep-D471L-F	AGTTGGTCTGGAGTATTGCTATGGAGGGCACAC	Mutagenesis
Rep-D471L-R	TACTCCAGACCAACTTGTGATGGGTTTCTTCC	Mutagenesis
18s-F	GGCCCTGTAATTGGAATGAGTC	qRT-PCR
18s-R	CCAAGATCCAACTACGAGCTT	qRT-PCR
Rep-WNVII-NS1-F	GGAGGTAGAGGACTTTGGATTTG	qRT-PCR
Rep-WNVII-NS1-R	GCACAGCCATGTTGTTCTTG	qRT-PCR
pET-In-Fusion F	TAATAGGCGGCCGCCTCG	Cloning
pET-In-Fusion R	GGATCCCTGGAAGTACAGGTTTTCG	Cloning
coNS3h-In-Fusion F	TACTTCCAGGGATCCATGGATGAGCCGATCCCG	Cloning
coNS3h-In-Fusion R	GGCGGCCGCCTATTAACGCTTTCCTGATGCAAAGTC	Cloning
coNS3h-D471E-F	GGTGGGGGAAGAATACTGTTACGGCGGGCAC	Mutagenesis
coNS3h-D471E-R	TATTCTTCCCCCACCTGCGAGGGATTG	Mutagenesis
coNS3h-D471N-F	GGTGGGGAACGAATACTGTTACGGCGGGCAC	Mutagenesis
coNS3h-D471N-R	TATTCGTTCCCCACCTGCGAGGGATTG	Mutagenesis
coNS3h-D471L-F	GGTGGGGCTGGAATACTGTTACGGCGGGCAC	Mutagenesis
coNS3h-D471L-R	TATTCCAGCCCCACCTGCGAGGGATTG	Mutagenesis
coNS3h-A287L-F	GGACGAGCTGCATTTTACTGATCCAGCAAGCATTG	Mutagenesis
coNS3h-A287L-R	AAATGCAGCTCGTCCATGACGAATAAGTTG	Mutagenesis
NS2b3-D471E-F	AGTTGGTGAGGAGTACTGTTATGGGGGGCACA	Mutagenesis
NS2b3-D471E-R	TACTCCTCACCAACTTGCGACGGATTTCT	Mutagenesis
NS2b3-D471N-F	AGTTGGTAACGAGTACTGTTATGGGGGGCACA	Mutagenesis
NS2b3-D471N-R	TACTCGTTACCAACTTGCGACGGATTTCT	Mutagenesis
NS2b3-D471L-F	AGTTGGTCTGGAGTACTGTTATGGGGGGCACA	Mutagenesis
NS2b3-D471L-R	TACTCCAGACCAACTTGCGACGGATTTCT	Mutagenesis
NS2b3-S135A-F	CTGGAACAGCAGGCTCACCAATAGTGGACAAA	Mutagenesis
NS2b3-S135A-R	AGCCTGCTGTTCCAGTGGGGAAGTCCA	Mutagenesis

1.3 Antibodies

Antibody	Vendor	Catalog number
Actin	Cell Signaling	4967
GFP	Cell Signaling	2956
WNV NS3	Genetex	GTX131955
WNV NS2b	Genetex	GTX132060
Strep	Abcam	Ab184224
anti-Rb-HRP	ThermoFisher Scientific	G-21234

2 Supporting Tables

Table S1: **Maintenance of octahedral coordination of Mg^{2+} within 2.5 Å for hydrolysis substrate and products bound WNV NS3h+ssRNA.**

**Atom Name**	**ATP**	**ssRNA+ATP**	**ssRNA+ADP+P_i**	**ssRNA+ADP**
$OG1_{T200}$	99.94%	99.98%	99.63%	99.84%
$OE2_{E285}$	99.94%	–	–	–
$O3G_{ATP}$	100%	–	–	–
$O2G_{ATP}$	–	100%	–	–
$O1B_{ATP}$	100%	100%	–	–
$O1B_{ADP}$	–	–	100%	100%
$O3_{P_i}$	–	–	100%	–
O_{W1}	100%	100%	100%	100%
O_{W2}	100%	100%	100%	100%
O_{W3}	–	100%	100%	100%
O_{W4}	–	–	–	100%

Table S2: **Occurrence probability of conserved contacts in different substrates bound WNV NS3 helicase models with ssRNA.** A 5 Å of cut-off distance is used between atoms of protein and ssRNA. The residue identity in WNV NS3h is similar to DENV.

**Motif**	**ZIKV**	**DENV**	**ssRNA**	**ssRNA+ATP**	**ssRNA+ADP+P_i**	**ssRNA+ADP**
**Ia**	P224	P223	99%	99%	99%	99%
**Ia**	T225	T224	99%	81%	99%	99%
**Ia**	R226	R225	99%	99%	99%	99%
**Ia**	–	V226	99%	99%	99%	99%
**II**	T290	T289	64%	8%	6%	63%
**II**	D291	D290	99%	99%	99%	99%
**IV**	P364	P363	7%	93%	8%	99%
**IV**	S365	S364	35%	99%	97%	99%
**IV**	V366	I365	12%	98%	99%	99%
**IV**	K367	R366	5%	56%	84%	95%
**IVa**	S387	S386	2%	99%	99%	9%
**IVa**	R388	R387	99%	99%	99%	99%
**IVa**	K389	K388	4%	63%	93%	8%
**V**	T409	T408	36%	99%	99%	98%
**V**	D410	D409	97%	99%	99%	4%
**V**	I411	I410	52%	99%	99%	98%

Table S3: **Occurrence probability of conserved contacts formed by WNV-NS3h with ATP, ADP+P_i and ADP in presence of ssRNA.** In ZIKV and DENV, the contacts are identified from crystal structure. In ZIKV, only ATP+ssRNA bound NS3h is considered (PDB:7V2Z), while in DENV all hudrolysis substrate and products bound structures (PDB:2JLV, 2JLY, 2JLZ) are taken into account. A 5 Å of cut-off distance is used between atoms of protein and ATP or ADP or P_i.

**Motif**	**ZIKV**	**DENV**	**ssRNA**	**ssRNA+ATP**	**ssRNA+ADP+P_i**	**ssRNA+ADP**
**I**	H195	H194	H194	61%	86%	3%
**I**	P196	P195	P195	99%	99%	97%
**I**	G197	G196	G196	99%	99%	99%
**I**	A198	A197	A197	99%	99%	99%
**I**	G199	G198	G198	99%	99%	99%
**I**	K200	K199	K199	99%	99%	99%
**I**	T201	T200	T200	99%	99%	99%
**I**	–	K201	R201	99%	67%	84%
**I**	–	R202	R202	97%	9%	49%
**Ia**	–	E230	E230	5%	32%	27%
**II**	E286	E285	E285	63%	98%	68%
**III**	–	A316	A316	8%	75%	–
**V**	M414	M413	M413	1%	99%	28%
**V**	G415	G414	G414	20%	99%	23%
**V**	–	A415	A415	88%	18%	56%
**V**	N417	N416	N416	92%	90%	94%
**VI**	Q455	Q456	Q456	8%	98%	–
**VI**	R459	R460	R460	99%	99%	97%
**VI**	R462	R463	R463	99%	99%	70%
**VI**	N463	N464	N464	5%	99%	97%

Table S4: **Significance of $E_{inter}^{NS3h-ADP}$ distributions with *t*-test.** We randomly selected 501 dataset from each of the two compared distributions. Same step of new random dataset is iterated for 1000K times. The reported absolute t-value is averaged over the iterations which is higher than the critical value (1.96) for 0.05 significance level of 1000 degrees of freedom (df)

**Compairing Systems**	***t*-value**
ssRNA+ATP and ssRNA+ADP+P_i	44.02(1.45)
ssRNA+ADP+P_i and ssRNA+ADP	77.61(2.0)
ssRNA+ATP and ssRNA+ADP	45.11(1.5)

Table S5: **Average interaction energy between NS3h protein and ssRNA, ADP+Mg^{2+} for all modelled hydrolysis states.** Standard deviation of last digit is provided in parentheses. See main text for error calculations. Units in kcal·mol^{-1}.

System	ssRNA	ADP	ADP+Mg^{2+}
**ssRNA**	-552.1(4)	–	–
**ATP**	–	-382.2(4)	-440.0(3)
**ssRNA+ATP**	-672.7(5)	-462.6(3)	-391.1(2)
**ssRNA+ADP+**P_i	-746.8(6)	-571.4(4)	-461.9(3)
**ssRNA+ADP**	-647.6(7)	-348.5(4)	-350.8(8)

Table S6: **MM-PBSA binding free energy of NS3h-ADP.** Units in kcal.mol^{-1} and standard-error of last digit is provided in parentheses.

**System**	$\Delta G_{binding,MMPBSA}$
**ssRNA+ATP**	-6.5(5)
**ssRNA+ADP+**P_i	-8.7(7)
**ssRNA+ADP**	37(1)

Table S7: **Cluster specific distances between key residues of MVIL.** To define the loop **MVIL** loop stattus we used sidechain distances of 461-464 and 464-471 residue pair. We chose terminal sidechain 'C' position of any residue to compute the distance. Parentheses vale denote standard deviation of means of last digit. Units is $\mathring{A}$. See text for valve status definition details.

**Residue Pair**	**WT**		**D471E**		**D471N**		**D471L**	
	$C1^W$	$C2^W$	$C1^E$	$C2^E$	$C1^N$	$C2^N$	$C1^L$	$C2^L$
461-464	5.07(2)	18.06(1)	12.33(2)	16.75(1)	12.55(2)	13.08(1)	7.81(1)	9.83(1)
464-471	19.44(1)	4.84(1)	16.41(3)	5.15(2)	12.63(3)	16.18(2)	20.72(1)	20.27(2)
Valve Status	**closed**	**open**	**open***	**open**	**gated**	**open***	**closed**	**open***

3 Supporting Figures

Figure S1: **Decomposition of NS3h-ADP binding enthalpy into protein residue.** Units in $kcal.mol^{-1}$

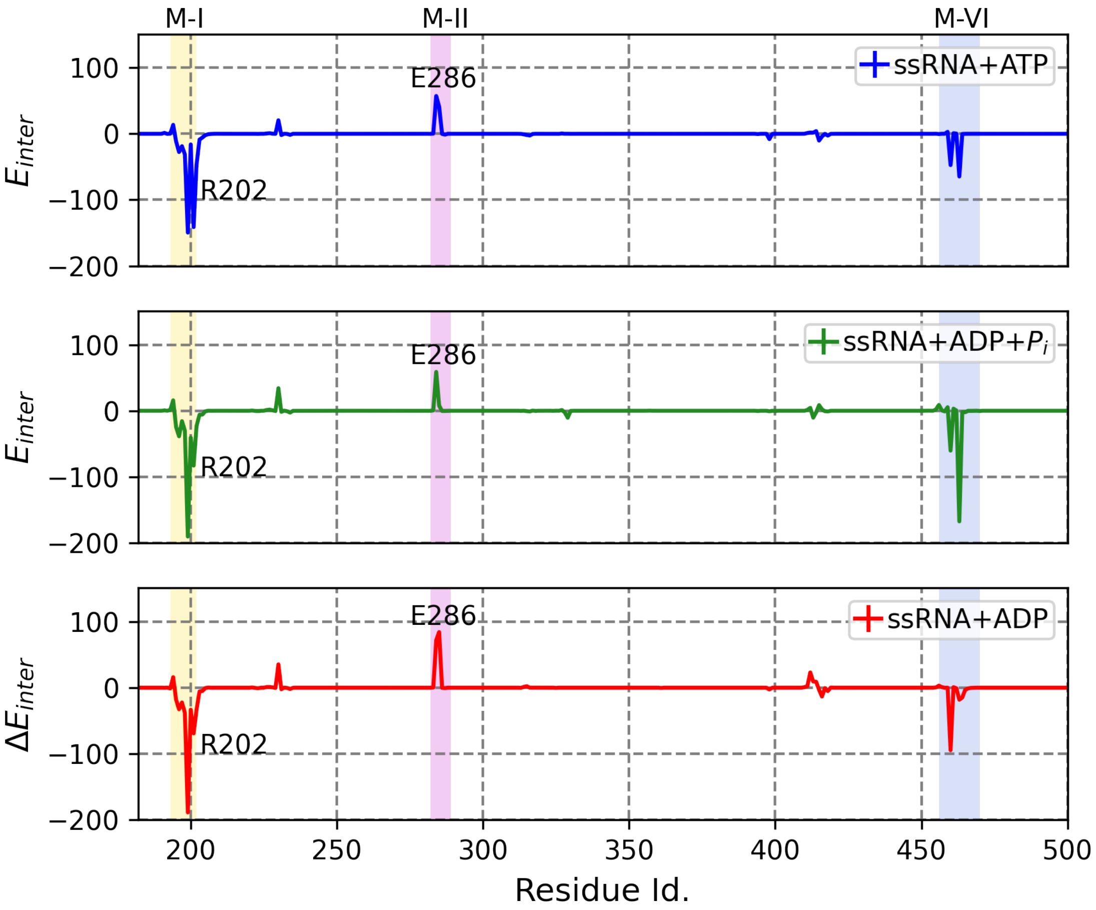

Figure S2: **Linear correlation of protein residues with R460 and R463.** Presented correlation is the Pearson's correlation of C_α atoms of protein. Averaged over the entire trajectory.

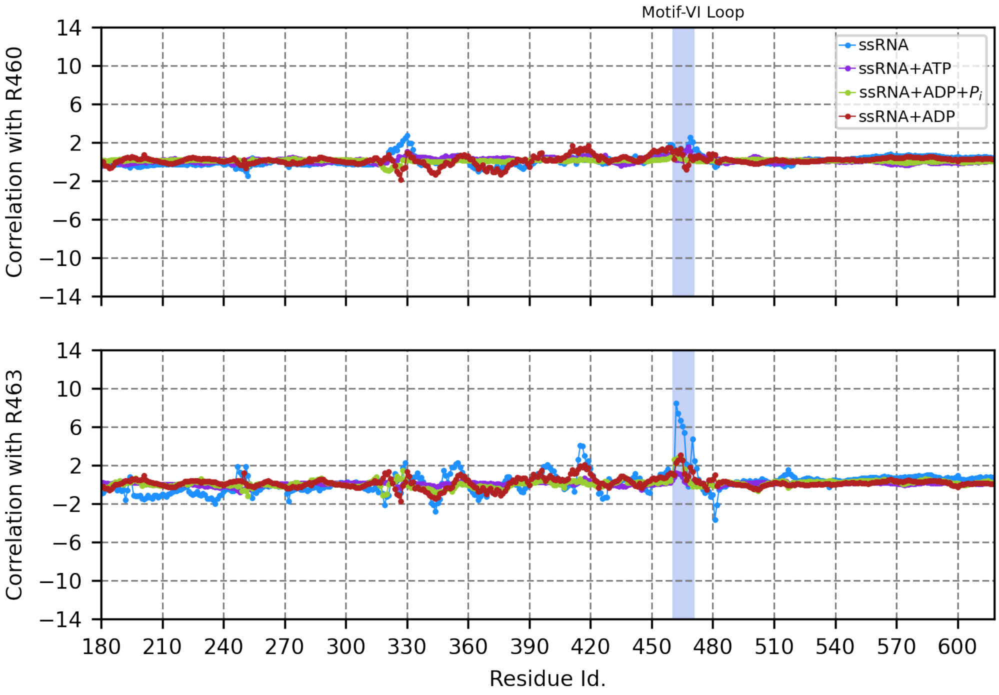

Figure S3: **Sequence and structure conservation of the *Orthoflavivirus* MVIL.** (a) Multi-sequence alignment. We performed multi-sequence alignment of TBEV, WNV, DENV4 and ZIKV NS3h protein sequences through CLUSTAL O (Omega) and highlighted the motif sequences. (b) NS3h structural alignment. The crystal structures of NS3h apo form is used for structural alignment and considered the C_α atoms for computing superposition parameters. In the figure only motifs regions are colored while remaining protein part colored in grey. The TBEV (PDB Id.: 7OJ4), WNV (PDB Id.: 2QEQ), DENV4 (PDB Id.: 2JLQ) and ZIKV (PDB Id.: 5JMT) colored in blue, red, green and orange respectively.

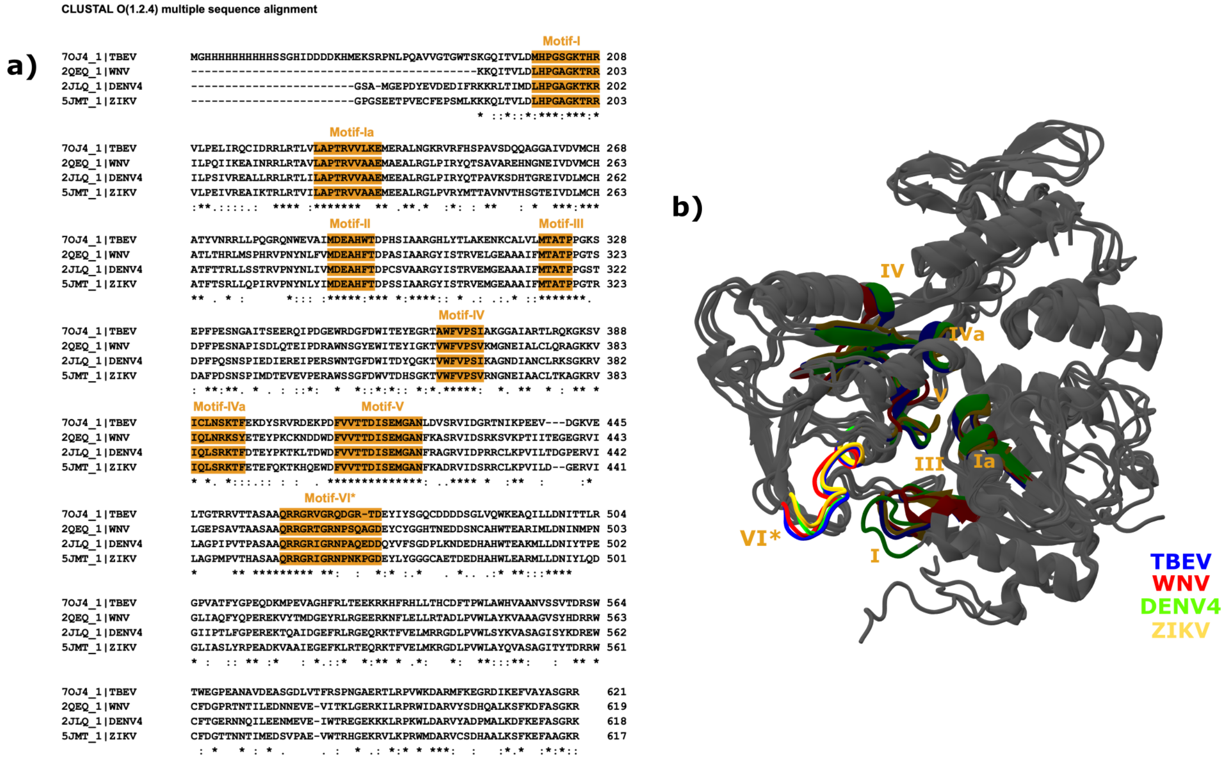

Figure S4: **Representation of MVIL clusters in presence of ATP only.** The clusters are predicted based on the Shape-GMM models of wild-type MVIL of hydrolysis states. The 'P' denotes probability of each clusters i.e. C1 and C5. At the bottom of each structural image, distances between the R461(NH2) or R464(NH2) with ATP phosphates is annotated.

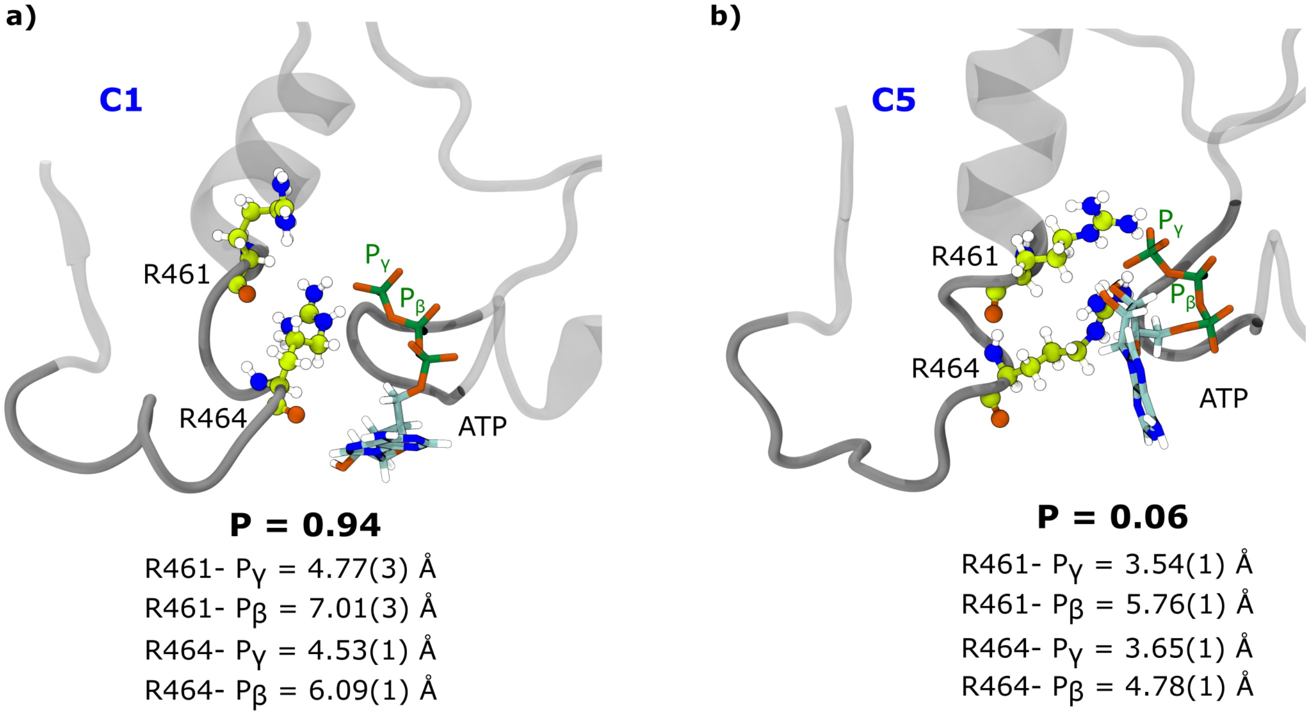

Figure S5: **Purification of WNV NS3h and ATPase Activity Assay.** a) Equal amounts (500ng) of purified recombinant NS3h was resolved on an SDS-PAGE gel and stained with GelBlue to visualize NS3h. Wild-type and mutant NS3h proteins display similar levels of purity. b) Plot of A650 over time, which measures the accumulation of free phosphate from ATP hydrolysis in the in vitro ATPase assay, at the indicated ATP concentrations. Slopes were calculated using the linear portion of the product accumulation curve and V0 was calculated using a standard curve of free phosphate.

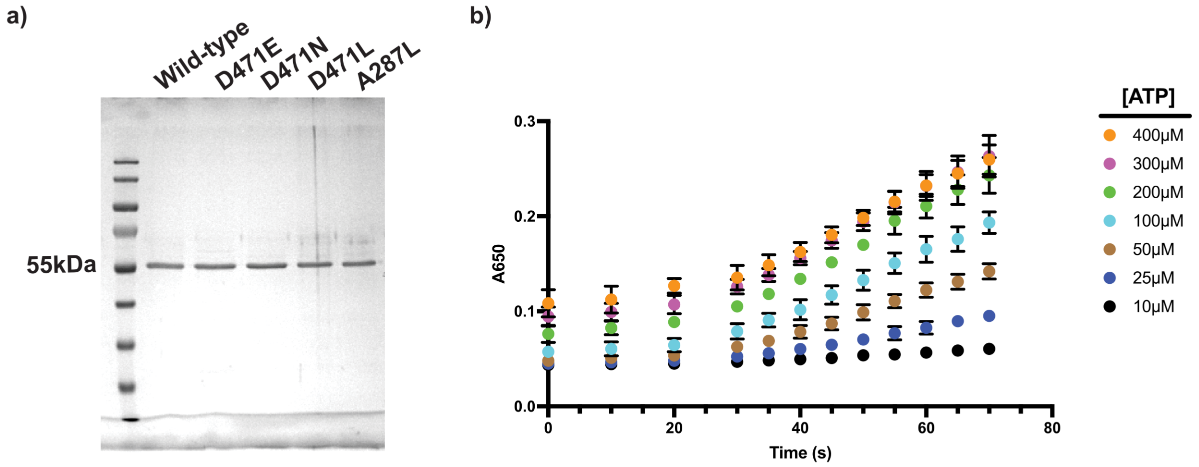

Figure S6: **Clustering of motif-VI loop ensembles sampled in the mutants of WNV NS3h apo state.** The log likelihood per frame as a function of clusters is plotted for each mutant with change of slope measure by 2nd derivative. The clustering is performed with ShapeGMM algorithm and the feature consists with positions of all heavy atoms of residues 461 to 472. Each training run is iterated for 5 times for each cluster.

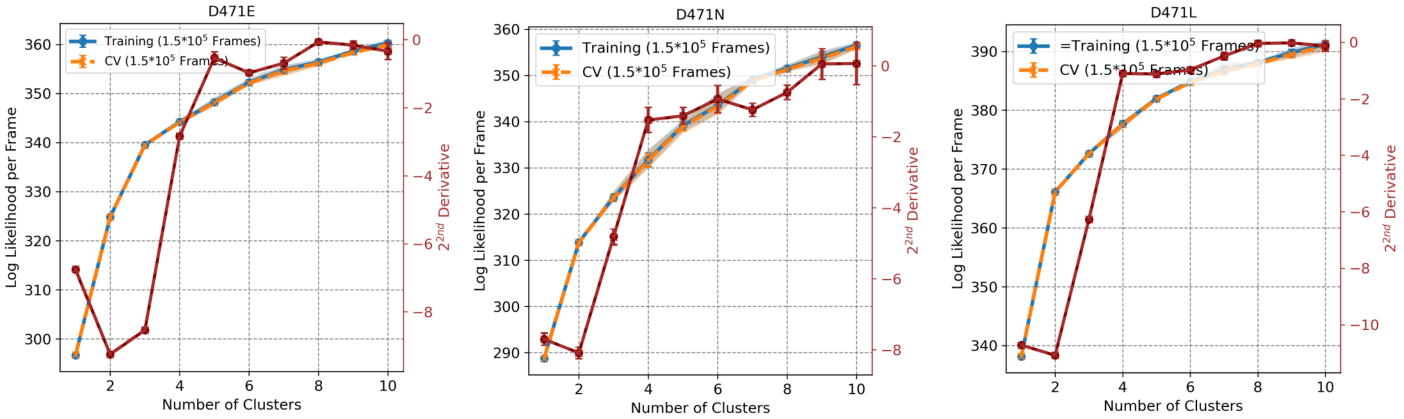

Figure S7: **Quantitation of GFP+ Cells and Protease Activity of NS3 Mutants**. a) Quantification of percent GFP+ cells for wild-type replicon and D471 mutants. Pol-, replicon encoding D668A substitution which prevents RNA-dependent RNA polymerase activity. Data shown are normalized to wild-type, n=3, statistics are one-way ANOVA with Dunnett's correction for multiple comparisons, performed on raw non-normalized data. ** p¡0.01, ***p¡0.005. b) Western blot of cells transfected with a tagged NS2b/3-Strep protease expression vector encoding wild-type NS2b/3, D471 substitutions, or a protease-dead S135A substitution. Cells were transfected with a pcDNA vector as a control

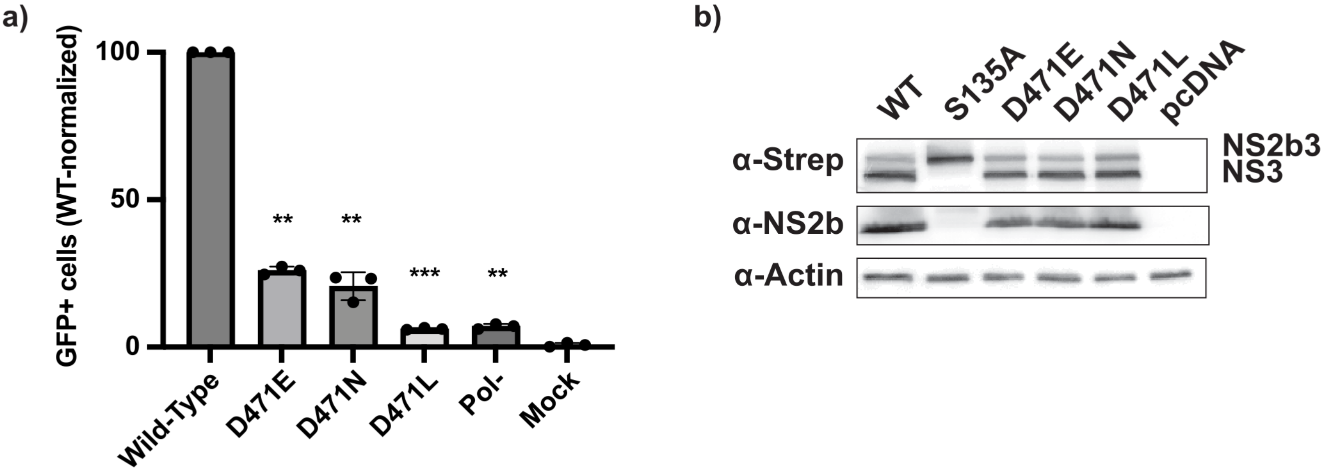

Supplement: gkae500_Supplemental_File [file gkae500_supplemental_file.pdf]
